# Type I and III interferons shape the retinal cytokine network and barrier function in an *in vitro* model of ocular toxoplasmosis

**DOI:** 10.3389/fimmu.2023.1148037

**Published:** 2023-05-02

**Authors:** Benjamin Geiller, Valentin Greigert, Caroline A. Hillenbrand, Chloé Gommenginger, Laetitia Beal, Julie Brunet, Denis Filisetti, Odile Villard, Julie Denis, Alexander W. Pfaff

**Affiliations:** ^1^ Institut de Parasitologie et Pathologie Tropicale, UR 7292 Dynamique des Interactions Hôte-Pathogène, Fédération de Médecine, Translationnelle, Université de Strasbourg, Strasbourg, France; ^2^ Service de Parasitologie et Mycologie Médicale, Hôpitaux Universitaires de Strasbourg, Strasbourg, France

**Keywords:** *Toxoplasma gondii*, retina, ocular immunology, interferons, inflammation, barrier function, tight junctions

## Abstract

**Introduction:**

The particularities of the ocular immune environment and its barrier protection in the context of infection are not well elucidated. The apicomplexan parasite *Toxoplasma gondii* is one of the pathogens successfully crossing this barrier and establishing chronic infection in retinal cells.

**Methods:**

As a first approach, we studied the initial cytokine network in vitro in four human cell lines: Retinal pigmented epithelial (RPE), microglial, astrocytic and Müller cells. Furthermore, we looked at the consequences of retinal infection on the integrity of the outer blood-retina barrier (oBRB). We particularly focused on the roles of type I and type III interferons, (IFN-β and IFN-λ). Especially IFN-λ is known for its significant role in barrier defense. However, its effect on the retinal barrier or *T. gondii* infection remains unexplored, unlike IFN-γ, which has been extensively studied in this context.

**Results and Discussion:**

Here, we show that stimulation with type I and III interferons did not limit parasite proliferation in retinal cells we tested. However, IFN-β and IFN-γ strongly induced inflammatory or cell-attracting cytokine production, whereas IFN-λ1 showed less inflammatory activity. Concomitant *T. gondii* infection influenced these cytokine patterns, distinctly depending on the parasite strain. Interestingly, all these cells could be stimulated to produce IFN-λ1. Using an in vitro oBRB model based on RPE cells, we observed that interferon stimulation strengthened membrane localization of the tight junction protein ZO-1 and enhanced their barrier function, in a STAT1-independent manner.

**Conclusion:**

Together, our model shows how *T. gondii* infection shapes the retinal cytokine network and barrier function, and demonstrates the role of type I and type III interferons in these processes.

## Introduction

The eye is, like the brain, protected by an efficient barrier system and a particular immune environment, which protects this fragile organ from damage by overwhelming inflammatory reaction (1). Pathogens entering into the eye have to manipulate both the barriers and the interaction between resident cells. The apicomplexan parasite *Toxoplasma gondii* uses brain and eye as a protective niche to achieve chronic infection and thus enhance transmission probability. This long-time persistence in retinal cells requires parasite-induced modification of the ocular immune environment. How the parasite enters the eye is still a subject of debate, despite recent advances (2, 3). In analogy to the blood-brain barrier which has been studied more intensively, the parasite reaches the retina via the outer or inner blood retinal barrier (oBRB/iBRB). The oBRB is made up by retinal pigmented epithelial (RPE) cells (4). The barrier function is provided by the highly modulable tight junctions, apico-lateral protein structures formed of trans-membranous proteins like occludin and claudins connected to the cell cytoskeleton through the zonula occludens (ZO) protein family (5). ZO-1 plays a central role in regulating tight junction integrity (6). Barrier permeability is affected by numerous mediators including interferons. In humans, three types of interferons are described. Type I essentially consists of IFN-α and IFN-β isoforms (7), Type II only of IFN-γ (8) and type III of four IFN-λ isoforms (9). Each interferon type binds to its specific heterodimeric receptor, signals through the JAK/STAT pathway (10) and subsequently induces the expression of interferon stimulated genes (ISGs) (11). The ISG expression can be redundant or specific to certain interferon types. Our previous studies showed the pathogenic roles of the resulting production of the inflammatory cytokines IL-6 and IL-17 (12, 13). These and many other studies demonstrated the central and protective role of IFN-γ in *T. gondii* induced immune response, by limiting parasite proliferation (14). Regarding type I interferons, the available results are less conclusive. While IFN-β dependent mechanisms, involving immune-related GTPases (IRGs), leading to parasitophorous vacuole destruction have been described in murine myeloid cells (15), no homologous mechanism has been found so far in humans. Type III interferons are mainly known for anti-viral activities, even if some recent studies looked at parasitic infection. Its role in *T. gondii* infection has not yet been addressed. Importantly, these interferons are mainly reputed for their role in barrier immune responses (16). This is reflected by the predominant expression of type III specific IFNLR1 receptor subunit in epithelial and endothelial cells, but also some myeloid and lymphoid cells, in contrast to the ubiquitously expressed type I interferon receptor (17). Importantly, IFN-λ stimulation results in a much weaker inflammatory reaction, due to a considerably lower density of its receptor on the cell surface (18). These specific features give to type III interferons a non-redundant unique role in barrier defense mechanisms, despite the common transduction pathway with type I interferons (19). The role of IFN-λ on the ocular barrier has been completely neglected so far. Of importance for our study, IFN-λ1 (IL-29) and IFN-β were shown to enhance blood-brain barrier function in a viral infection model. However, the exact mechanism by which type I and III interferons are enhancing barrier function stays elusive. Here, we conducted *in vitro* studies to address the role of type I and III interferons in this process, using various human non-neuronal retinal cell types, namely microglia, astrocyte, Müller and RPE cell. We showed that, while type I and III interferons do not control parasite proliferation in these cells, they do shape secretion of inflammatory cytokines and chemokines, interacting with *T. gondii* infection. Importantly, interferons and infection modulate ZO-1 tight junction organization in differentiated RPE cells, and thus barrier permeability, in an STAT1 independent manner.

## Materials and methods

### Cell lines and parasites

Retinal pigmentary epithelial RPE cells ARPE-19 (ATCC) were cultivated in DMEM/F-12K Glutamax (Gibco) 10% SVFi (Gibco) Penicillin/Streptavidin (Hyclone). Astrocytes U-118MG (ATCC) were cultivated in DMEM 4.5g/L L-Glutamine (Lonza), 10% SVFi, P/S. Müller MOI-M1 (UCLB,UK) and Microglia HMC3 (ATTC) were cultivated in DMEM (Gibco), 200mM glutamate (Gibco), 10% SVFi, P/S. All cell incubations were performed at 37°C, 5% CO_2_.

The *Toxoplasma gondii* strains RH and Me49 were obtained from the French Biological Resource Center Toxoplasma (CRB Toxoplasma; Laboratoire de Parasitologie, CHU Reims, Reims, France). Tachyzoites of the RH strain were maintained by weekly passages in Swiss-Webster mice, for use in medical diagnostic tests. The Me49 strain was maintained in Vero (ATCC) cell cultures.

### Flow cytometry

Cells were harvested with 0.05% trypsin EDTA and incubated in 37°C and 5% CO_2_ under agitation during 24 hours. Cells were then counted and 1x10^6^ cells per conditions prepared for labeling. Cell Fc-receptors were blocked using Fc binding inhibitor (Invitrogen) and cells were labeled with 5µg/mL mouse AlexaFluor488 anti-IFNLR1 antibody (R&D Systems) or AlexaFluor488 isotype control antibody (Invitrogen, A21467) in FACS buffer (1xPBS, 5% *SVFi*, 0.1% NaN_3_ sodium azide*).* Cells were analyzed using an Attune cytometer (ThermoFisher) with following gating strategy: First, cell debris were eliminated and viable cells were gated using FSC-A/SSC-A dot plot, then doublets were eliminated using SSC-A/SSC-H dot plot. Voltage was set with isotype control sample and maintained for all analysis.

### Impact of interferon stimulation on parasite proliferation

Cells were plated in 24 well culture plates (TPP), incubated for 24 hours with 20ng/mL of IFN-β, IFN-γ, IFN-λ1 (Peprotech) or control PBS BSA 0.1% and then infected with *T. gondii* RH or Me49 strain at a MOI of 1:10. To allow parasite invasion, the cultures were first incubated for 2h (RH) or 3h (Me49), when half of the cell cultures were harvested. The remaining cultures were washed with PBS to remove extracellular parasites and further incubated in medium plus interferons for 24h (RH) or 48h (Me49). Cells were harvested with 0.05% Trypsin-EDTA. DNA was extracted using the QIAamp DNA tissue minikit (Qiagen, France), eluted with 200μl elution buffer, and stored at −20°C. Real-time PCR specific for the TgB1 sequence of *T. gondii* (**Table 1**) was performed on a CFX light cycler using SsoAdvanced SYBRGreen supermix (Biorad). Parasite quantification was done using external standard samples with known parasite concentrations.

**Table 1:** PCR Primers.

Target	Forward	Reverse
*RPE65*	5’-GATCTCTGCTGCTGGAAAGG-3’	5’-TGGGGAGCGTGACTAAATTC-3’
*ZO-1*	5’-AGCCATTCCCGAAGGAGTTG-3’	5’-ATCACAGTGTGGTAAGCGCA-3’
*OCLN*	5’-GGAGGACTGGATCAGGGAT-3’	5’-TCAGCAGCAGCCATGTACTC-3’
*ITGB5*	5’-CGGGGACAACTGTAACTGCT-3’	5’-ACGCAATCTCTCTTGGTGCT-3’
*MERTK*	5’-AGACTTCAGCCACCCAAATG-3’	5’-GGGCAATATCCACCATGAAC-3’
*BEST1*	5’-CCCGAAAATCACCTCAAAGA-3’	5’-GCTTCATCCCTGTTTTCCAA-3’
*GAPDH*	5’-AGCAATGCCTCCTGCACCACCAAC-3’	5’-CCGGAGGGGCCATCCACAGTCT-3’
*TgB1*	5’-GGAACTGCATCCGTTCATGAG-3’	5’-TCTTTAAAGCGTTCGTGGTC-3’

### ARPE-19 cell line differentiation

Cells were differentiated according to the protocol described by Hazim et al. (20). Briefly, cells were cultivated in T75 flask for two weeks with MEM-Nicotinamide medium: 1% N1 (Invitrogen), 1% SVFi, 1% P/S, 0.25mg/mL Taurine (Sigma-Aldrich), Hydrocortisone 20ng/mL (Sigma-Aldrich), Triiodo-thyronine 0.013 ng/mL (Sigma-Aldrich) and 10mM nicotinamide (Sigma-Aldrich). After 3 times 10min TrypLE (Gibco) treatment, the remaining cells were placed on 0.4µM PET transwell inserts (Falcon, 353095) in 24-well-plates, coated with natural mouse laminin (Invitrogen). Cell medium was changed 3 times per week during 8 weeks before use. Differentiation was verified using RT-PCR analysis of markers indicating primary like cell phenotype. Cells on transwells were harvested every week during 8 weeks using Nucleozol (Macherey-Nagel, MN). RNA was extracted with TriZol reagent, according to the manufacturer’s recommendations. Then, 20ng of RNA was reverse transcribed to cDNA using Qscript reagent (VWR). Real-time PCR specific for the BEST1, RPE65, OCLN, MERTK, ZO-1, ITGB5 and housekeeping gene GAPDH mRNA (**Table 1**) was performed on CFX light cycler and SSoAdvanced SybrGreen (Biorad).

### Confocal microscopy

Differentiated ARPE-19 cultures on inserts were stimulated with 20ng/mL of IFN-β, IFN-γ or IFN-λ1, or infected with *T. gondii* RH strain at a MOI of 1:1, as schematically shown in **Figure S1F**. Cells were first fixed with PBS 4% paraformaldehyde for 15 minutes and permeabilized with 0.1% Triton X-100 for 10 minutes. Then, samples were labeled successively with 5µg/mL mouse anti-ZO-1 (Invitrogen, 33-9100) and goat anti-mouse IgG Alexa Fluor 555 (Invitrogen, A32727), or with 5µg/mL mouse anti-IFNLR1 Alexa Fluor 488 (RDsystems, FAB5260G). For cells cultivated on transwells, the membranes were first cut out and then mounted on microscopy slide with Prolong Gold (Invitrogen). Otherwise, cells were cultivated on round cover slides and then similarly mounted.

Images were taken with a Zeiss LSM 800 Airyscan confocal microscopy system. Settings were defined at the beginning of each experiment with the first control sample and maintained for all other samples of the same replicate. Image post treatment and measurements were made with FIJI software. For figures, minimum brightness was set to 100. For all measurements, raw images were used. The measurement of tight junction fluorescence was realized as follows (https://imagej.net/imaging/segmentation): First, auto threshold (method=default white) was applied, then the image was converted to mask and copied by the tool “Create selection”. This selection was then restored on the raw image and the mean gray values and area data were gathered. The same protocol was applied to all images.

### Supernatant cytokine dosage

RPE cells, Müller cells, astrocytes and microglial cells were plated in 24 well culture plates (TPP) for 24h. Then, they were stimulated with 20ng/mL IFN-β, IFN-γ or IFN-λ1, or infected with *T. gondii* RH or Me49 strain (MOI 1:1) for 20h. Cell culture supernatants were recovered and 14 cytokines were dosed (IL-1β et α, IL-23, IL-12p70, TNF-α, IL-6, IFN-β, IL-29 (IFN-λ1), IL-10, IL-17A, CXCL10, CXCL8, CXCL9, CCL2) with a ProcartaPlex (ThermoFisher) kit on an Magpix Luminex (ThermoFisher) according to manufacturer’s recommendations.

### STAT1 immunoblotting

RPE cells were plated in 10mL petri dishes (TPP) for 24h. Then, they were treated with different concentrations of fludarabine (Sigma-Aldrich) for 24h before 20ng/mL IFN-β, IFN-γ or IFN-λ1 stimulation. After 1h stimulation for IFN-γ, IFN-β and 3h stimulation for IFN-λ1 cells were washed in 1xTBS and harvested using RIPA buffer. Lysates were then denatured at 100°C for 5 minutes in Laemmli buffer (Biorad) and protein separated in 10% SDS polyacrylamide gel at 150V for 1h using an Invitrogen mini gel tank and blot module (Invitrogen). Proteins were then transferred on a polyvinylidene difluoride PVDF membrane at 250mA for 1h. Membranes were then blocked 2h with TBS bovine serum albumin (BSA) 5% at room temperature. Membranes were then incubated with primary STAT1 (Invitrogen, AHO0832) at 500ng/mL, Tyr701 PhosphoSTAT1 at 500ng/mL (Invitrogen, 33-3400) and β-actin at 50ng/mL (Invitrogen, A5441) antibodies over night at 4°C. Finally, membranes were incubated 2h at room temperature with 125ng/mL sheep anti-mouse IgG HRP (GE healthcare, NA931V) secondary antibodies and visualized with clarity western ECL substrate (Biorad) on ChemiDoc MP (Biorad). Band intensity was then analyzed using FIJI gel tools.

### FITC dextran permeability assay and electric resistance

After the differentiation protocol, RPE cells were pre-incubated for 24h with 250µM fludarabine or in MEM-Nic medium, as indicated. Then, cells were stimulated with 20ng/mL of IFN-β, IFN-γ or IFN-λ1, or infected with *T. gondii* RH strain at a MOI of 1:1, as schematically shown in **Figure S1F**. For the FITC dextran assay, 1mg/mL 70kDa fluoresceine dextran (Invitrogen) in DMEM/F-12K without phenol red (Gibco) was placed in the upper transwell chamber. After an incubation time of 48h for interferon stimulated condition and 24h for infected conditions, 50µL medium was taken in the lower chamber. These time points had been established in preliminary experiments. The FITC mean fluorescence intensity (MFI) was measured at 521nm wavelength using a Varioskan Lux (ThermoFisher). Electrical resistance was measured at 0h, 24h and 48h with a calibrated EOM2 probe (World precision instruments) at 37°C and expressed as TEER [Ohm x cm^2^].

### Statistical analysis

Statistical analysis was performed on Graphpad Prism v8.0. Normality was confirmed for all groups with Shapiro-Wilk normality test at α=5%. To compare groups, unpaired t-tests were performed, with statistical significance considered at p < 0.05. Considered sample sizes (n) and replicate numbers are mentioned below each figure.

## Results

### All cell lines in this study express the type III interferon specific subunit IFNLR1

**Figure 1:**
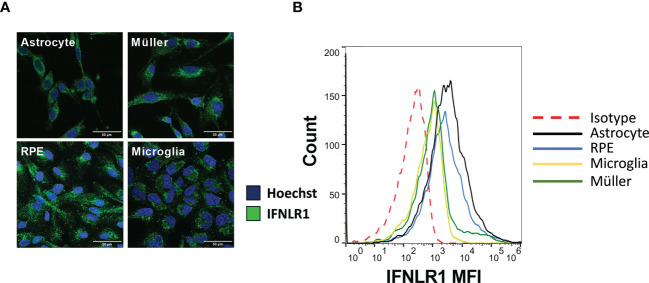
The four cell lines express IFNLR1 receptor subunit. **(A)** Confocal microscopy image of RPE, astrocytes, microglia and Müller cells. Cells were plated on cover slides for 24h. The cells were then fixed, permeabilized and the IFNLR1 subunit and cell nuclei labeled with AlexaFLuor488 and Hoechst 33342, respectively. **(B)** Flow cytometry assay for IFNLR1 receptor subunit. Cells were harvested by trypsination and placed 24h under agitation to restore membranous receptor expression. Finally, they were labeled and analyzed as described in Material and Methods.

Unlike type I and II interferon receptor subunits, the IFNLR1 subunit, necessary for IFN-λ signaling, is not ubiquitously expressed. Therefore, we first verified their expression in the four human cell lines used in this study by confocal microscopy and flow cytometry. The microscopy images clearly showed an expression by all cell types (**Figure 1A**). Flow cytometry analysis confirmed these results (**Figure 1B**). Nevertheless, expression of the receptor was relatively low, as shown by the small difference with the isotype control. Astrocytes and RPE cells appeared to have a higher receptor expression than the other cell lines tested, but also here, the difference was small and not visible on microscopy images. We conclude that all studied cell types express the IFNLR1 subunit at relatively low levels.

### Type I and III interferons do not limit *Toxoplasma gondii* proliferation

**Figure 2:**
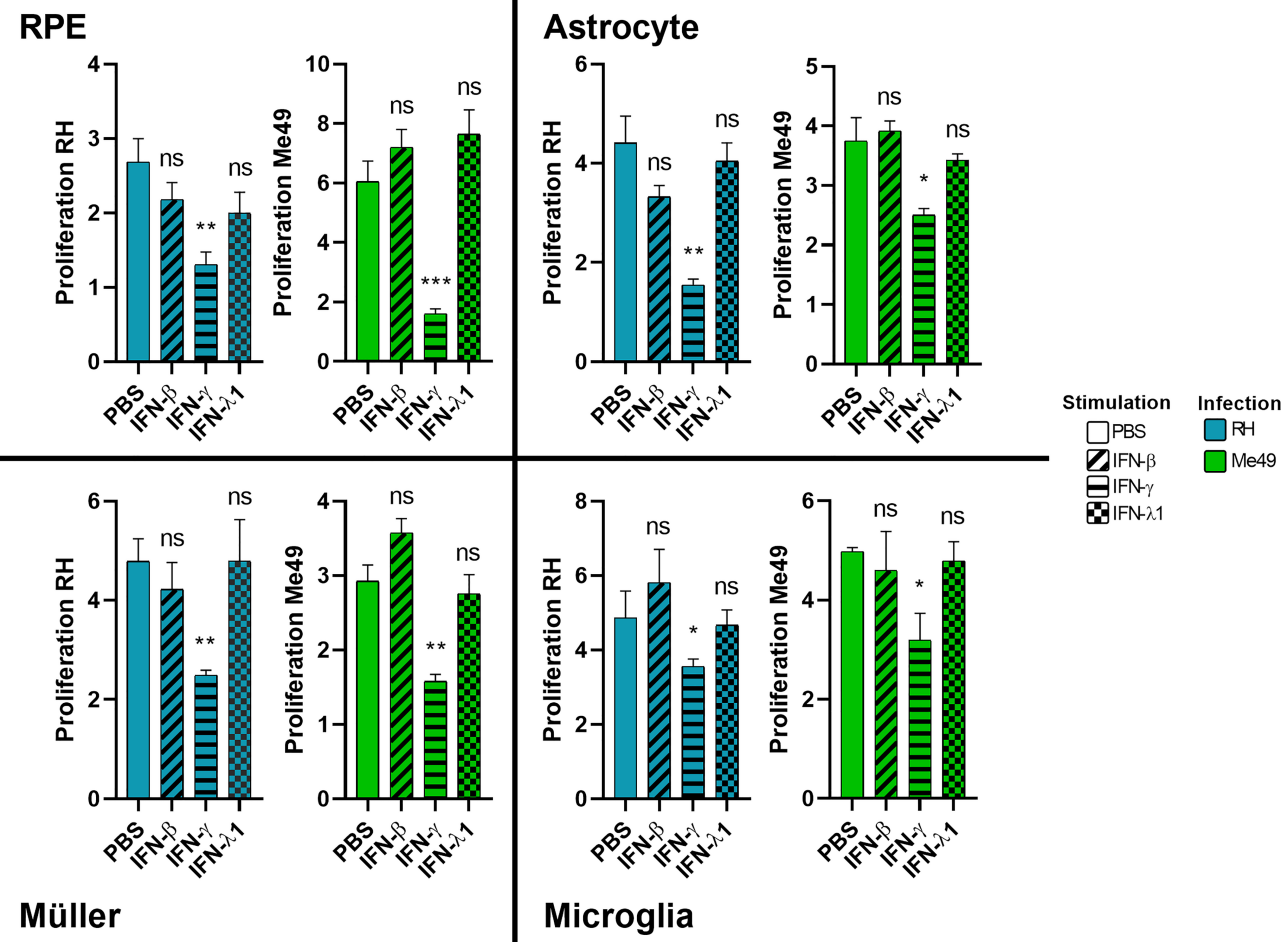
Type II, but not type I and III interferons inhibit parasite proliferation. Cells were stimulated for 24 hours with 20g/mL of indicated interferons before being infected by RH or Me49 *T. gondii* strain at a MOI of 1:10. Proliferation is calculated as fold changes between parasite numbers at 2h p.i (RH) and 3h p.i (Me49) (invasion), compared to 24hpi and 48hpi, respectively (proliferation), as determined by qPCR. Results are expressed as means ± SEM, n=4. The experiment was repeated 3 times independently with similar results. ns: non significative, * *P*<0.05, ** *P*<0.01, *** *P*<0.001, compared to PBS control.

The role of interferon-gamma in controlling *T. gondii* proliferation is established. The role of type I interferons but also of the most recently discovered type III interferons has yet to be elucidated. Therefore, we studied the effect of stimulation of human cells with these interferons on parasitic proliferation by real-time quantitative PCR. As such control may vary between parasite strains, proliferation was assessed for the virulent RH strain as well as the avirulent Me49 strain. The results in **Figure 2** show that IFN-γ significantly inhibited the parasitic proliferation of RH and Me49 strains in all studied cell types. This reduction was about 50%, compared to the PBS control. By contrast, neither IFN-β, nor IFN-λ1, exerted significant inhibition of parasite proliferation in any cell type or by any strain used. No significant difference in parasite number was observed at 2h p.i. or 3h p.i., indicating no effect of IFN-β, IFN-γ and IFN-λ1 on parasitic cell invasion (not shown). These results highlight that neither type I IFN-β nor type III IFN-λ1 can directly limit parasite invasion or proliferation. Nevertheless, to verify indirect effects, we next studied their abilities to act on cytokine secretion profiles of human retinal cell lines in response to infection.

### Interferons strongly shape ocular cytokine secretion

**Figure 3:**
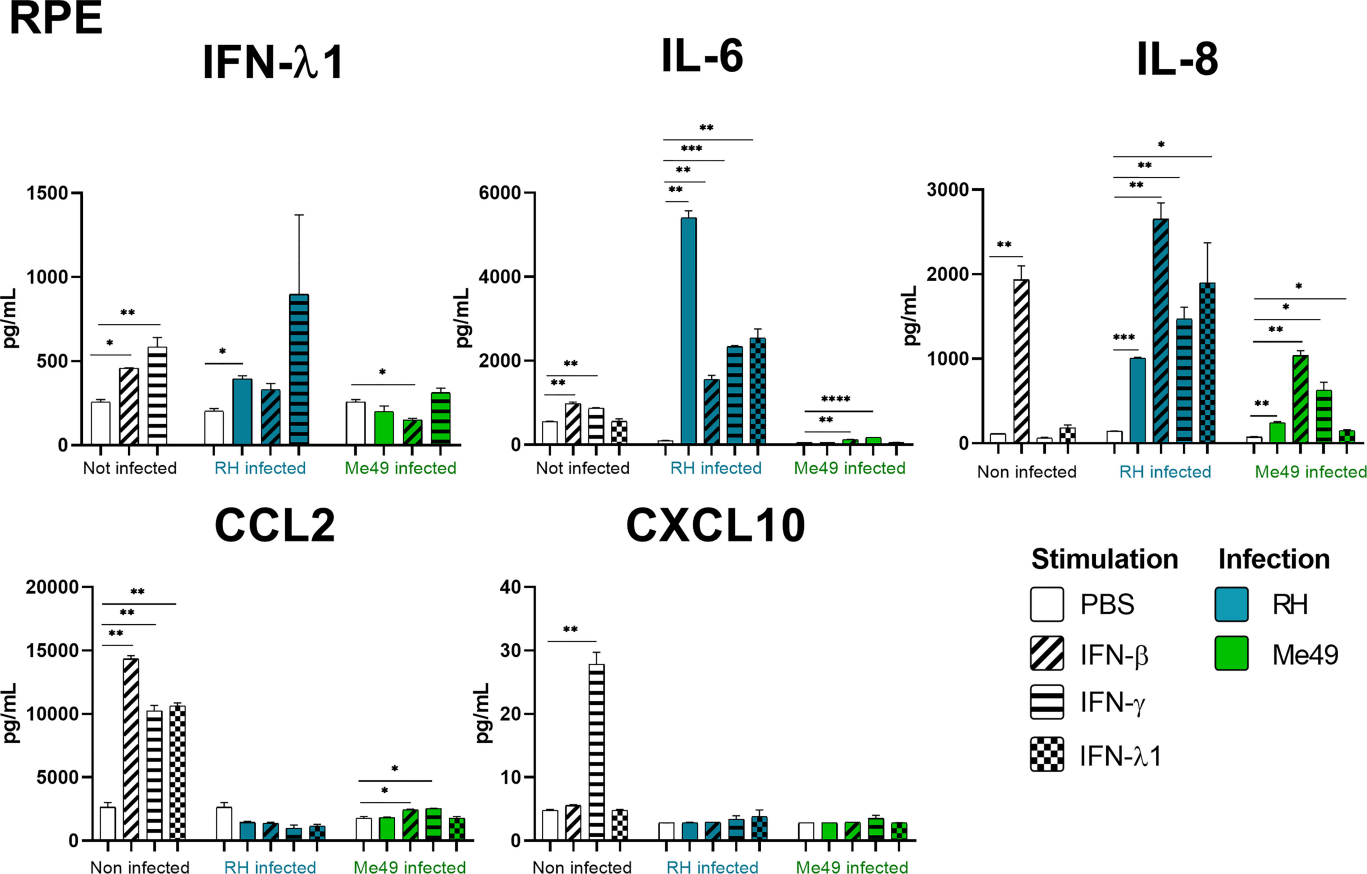
RPE cytokine secretion is differentially modified in a parasite strain and interferon type dependent manner. Cells were stimulated with 20ng/mL IFN-β, IFN-γ, IFN-λ1, as indicated, and/or infected with *T. gondii* RH or Me49 (MOI 1:1) for 20h. Cell culture supernatant was recovered and 14 cytokines were dosed (IL-1β et α, IL-23, IL-12p70, TNF-α, IL-6, IFN-β, IL-29, IL-10, IL-17A, CXCL10, CXCL8, CXCL9, CCL2). Only cytokines showing levels ≥ 10pg/mL in at least one condition are shown. Each group, non-infected (white), RH infected (blue) and Me49 infected (green) is compared to its independent non-infected/non-stimulated control. Results are means ± SEM of 2 independent experiments. * *P*<0.05; ** *P*<0.01; *** *P*<0.001; **** *P*<0.0001.

**Figure 4:**
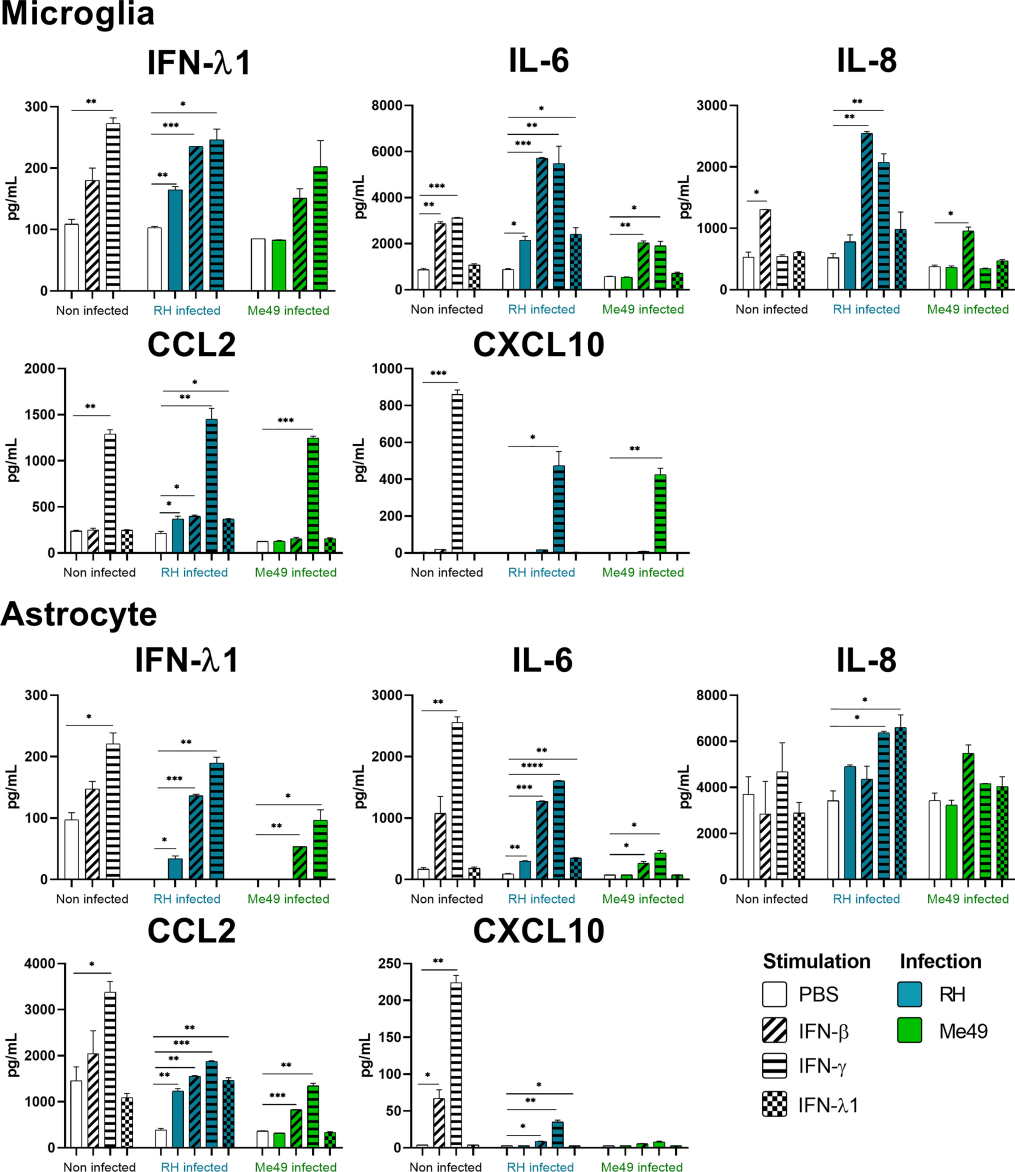
Microglia and Astrocyte cytokine secretion is differentially modified in a parasite strain and interferon type dependent manner. Cells were stimulated with 20ng/mL IFN-β, IFN-γ, IFN-λ1, as indicated, and/or infected with *T. gondii* RH or Me49 (MOI 1:1) for 20h. Cell culture supernatant was recovered and 14 cytokines were dosed (same as in **Figure 3**). Results are means ± SEM of 2 independent experiments. Each group, non-infected (white), RH infected (blue) and Me49 infected (green) is compared to its independent non-infected/non-stimulated control. Results are means ± SEM of 2 independent experiments. * *P*<0.05; ** *P*<0.01; *** *P*<0.001; **** *P*<0.0001.

**Figure 5:**
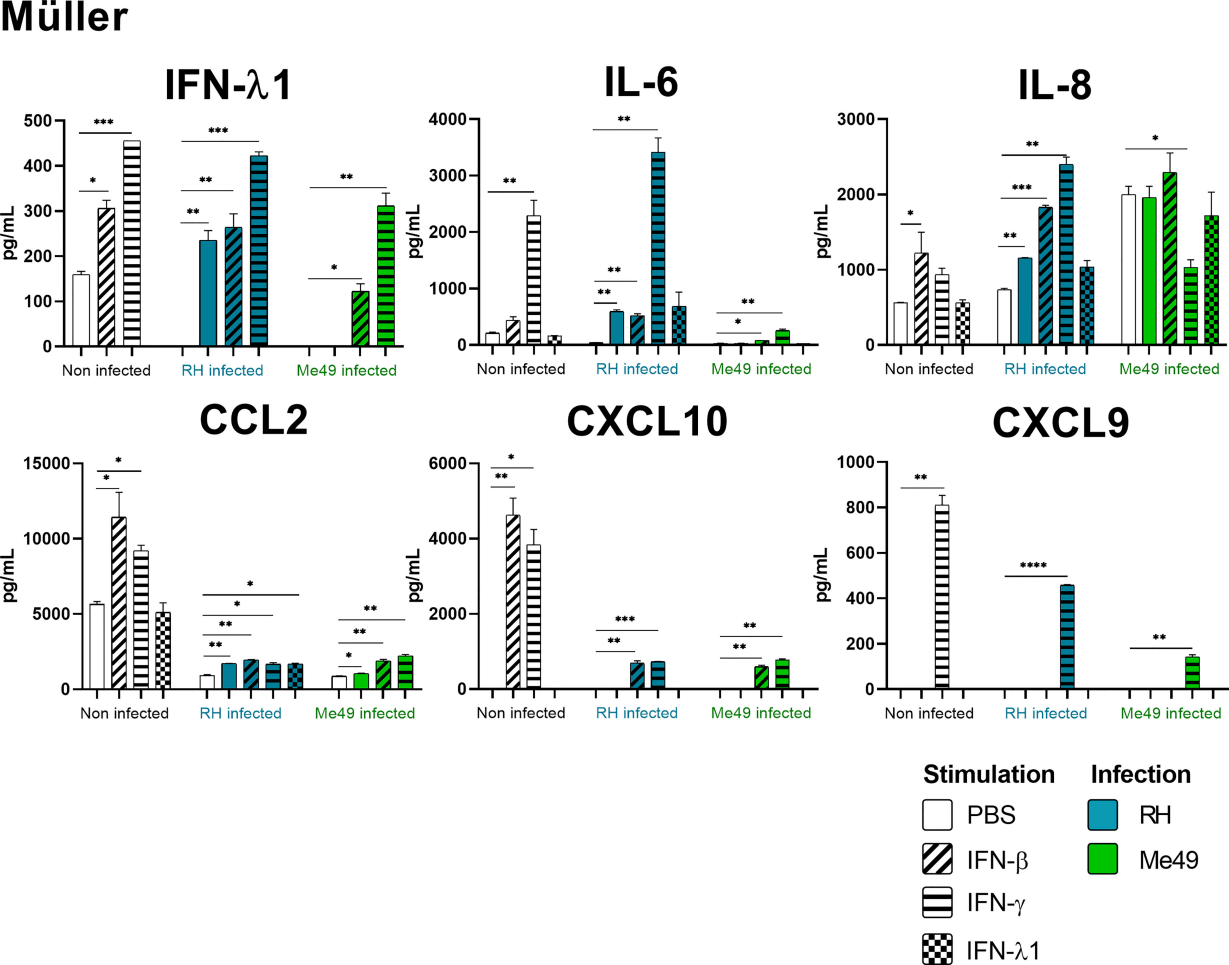
Müller cytokine secretion is differentially modified in a parasite strain and interferon type dependent manner. Cells were stimulated with 20ng/mL IFN-β, IFN-γ, IFN-λ1, as indicated, and/or infected with *T. gondii* RH or Me49 (MOI 1:1) for 20h. Cell culture supernatant was recovered and 14 cytokines were dosed (same as in **Figure 3**). Each group, non-infected (white), RH infected (blue) and Me49 infected (green) is compared to its independent non-infected/non-stimulated control. Results are means ± SEM of 2 independent experiments. * *P*<0.05; ** *P*<0.01; *** *P*<0.001; **** *P*<0.0001.

Our results show that IFN-γ, but not IFN-β and IFN-λ1 control parasite proliferation in the four cell types studied. We then measured the cytokines induced in response to stimulation with these interferons, with or without *T. gondii* infection in these cells, using a 14-plex Luminex bead-based assay (**Figures 3**–**5**). Remarkably, secretion of IFN-λ1 was significatively enhanced by IFN-γ in all cell types and by IFN-β in RPE and Müller cells. The highest levels were measured in RPE and Müller cell cultures with 585 pg/mL and 450 pg/mL, respectively. The inflammatory cytokine IL-6 was significantly up-regulated by IFN-β and IFN-γ in all cell types, with the exception of IFN-β stimulated astrocytes and Müller cells, where significance was not reached. By contrast, IFN-λ1 stimulation did not induce IL-6 production in any cell type. The equally inflammatory cytokine IL-8 showed already very high baseline expression in Astrocytes, which was not further enhanced by interferon stimulation. Microglial and Müller cells showed intermediate baseline secretion, which was further enhanced by IFN-β stimulation. In RPE cells, the lower IL-8 baseline secretion was substantially amplified in response to IFN-β. IFN-γ and IFN-λ1 stimulation did not influence IL-8 expression in any cell type. The chemokine CXCL10 (IFN-γ induced protein 10) was, as expected, induced by IFN-γ in all cell types, but also by IFN-β in astrocytes and Muller cells. The chemokine CCL2 (MCP-1) was strongly induced by IFN-γ in all cell types, by IFN-β in Müller and RPE cells and IFN-λ1 in RPE cells only. Again, IFN-λ1 stimulation resulted mostly in lower levels than the other interferons. Lastly, CXCL9 (MIG) was only detected upon IFN-γ stimulation, and only in Muller cells. All other cytokines tested were not, or very lowly expressed. To summarize, we first confirmed that all cell types are effectively responding to all three types of interferons. Then, we showed that IFN-λ1 is constitutively secreted by Astrocytes, RPE and Müller cells and further enhanced by interferon stimulation in all cell types. Finally, the inflammatory actions of type I and II interferons were not observed with type III interferons. Next, we looked at cytokine secreted in response to infection by two different parasite strains.

### *Toxoplasma gondii* RH infection but not Me49 induces pro-inflammatory cytokine secretion

As next step to study cellular interaction during *T. gondii* infection, we looked at cytokine secretion in cell culture supernatants of infected microglia, astrocytes, RPE and Müller cells. Since different parasite strains are known to induce very different cellular reactions, secretion profiles of human ocular cell lines in response to infection by *T. gondii* RH and Me49 strains were investigated (**Figures 3**–**5**). With RH infection, we observed significantly increased secretions of IFN-λ1 and IL-6 in all cell types, whereas secretion of CCL2 was significatively increased in microglia, astrocytes and Müller cells, but not RPE cells. RH infection also significantly increased secretion of IL-8 in RPE and Müller cells, but not in microglia nor in astrocytes, here probably due to the high baseline secretion. Similarly, RPE and Müller cells were the ones that secreted the most IFN-λ1 (around 400 pg/mL and 250 pg/mL respectively) in response to RH infection. Surprisingly, RPE cells also secreted the most IL-6 (up to 5400 pg/mL) in response to infection. Taken together, this points to a central role of RPE cells in immune response to *T. gondii* infection. In sharp contrast, the impact of Me49 infection was limited to moderately increased secretion of IL-8 by RPE cells and CCL2 by Müller cells.

### *Toxoplasma gondii* infection modifies interferon-mediated cytokine secretion

Our previous results show local production of IFN-λ1. Moreover, high levels of type I and II interferons are described during *in vivo* ocular infections. Therefore, we next studied the impact of the three types of interferons on the cytokine secretion profiles of infected cells (**Figures 3**–**5**). Notably, we observed synergistic effects for IL-6 and IL-8 secretion in microglia cells by concomitant RH infection and interferon stimulation. Again, this effect was generally less pronounced for IFN-λ stimulation. Interestingly, CXCL10, which was not spontaneously expressed and not induced by infection, showed considerably less interferon-induced expression in RH and Me49 infected cells. The same observation was made with the related chemokine CXCL9, only expressed in Müller cells. These results suggest that, even if type I and III interferons do not control *T. gondii* proliferation in retinal cells, there is a strong interaction between infection and interferon induced cytokine secretion, shaping the inflammatory environment in the retina during ocular toxoplasmosis. Taken together, these cytokine secretion profiles show that retinal resident cells secrete important amounts of chemokines, type III interferons and inflammatory cytokines in response to both infection and interferon stimulation. Importantly, induction of inflammatory cytokines was not observed with IFN-λ1 stimulation. These results raise the question of the impact of interferons on barrier integrity and particularly of type III interferons already described as being involved in such situations.

### Interferons and *Toxoplasma gondii* infection cause ZO-1 mediated tight junction reorganization in a human outer blood-retina barrier model

**Figure 6:**
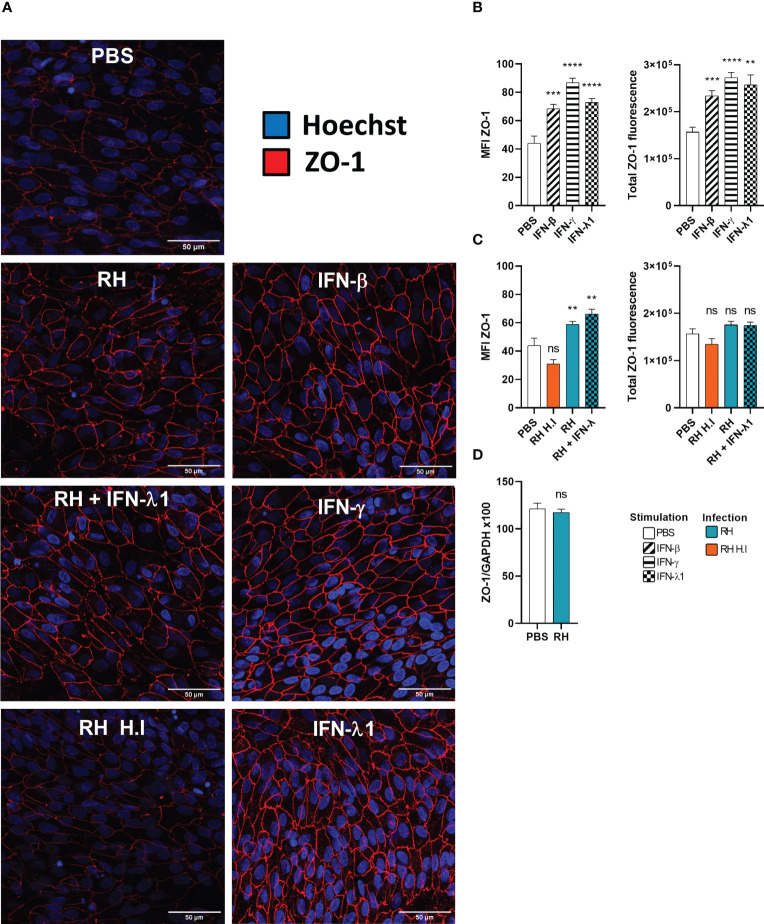
Interferons and infection modulate ZO-1 tight junction organization. **(A)** Confocal microscopy images of the outer blood-retina barrier model showing the effect of interferon stimulation and toxoplasmic infection on ZO-1 expression and localization. ARPE-19 RPE cells were differentiated for 8 weeks on laminin coated transwells. Then, they were treated with 20ng/mL IFN-β, IFN-γ or IFN-λ1 and/or infected at a MOI of 1:1 with live or heat-inactivated (H.I.) *T. gondii* RH strain for 14h. The ZO-1 protein was labeled with AlexaFluor 555 and cell nuclei with Hoechst 33342. **(B)** and **(C)** Fluorescence intensity analysis. MFI = Mean fluorescence intensity. Total ZO-1 fluorescence = MFI of the junction area x junction area. Results were expressed as means ± SEM, n=12 (4 transwells per condition and 3 images per transwell). The experiment was repeated 3 times independently with similar results. ns: non significative, * *P*<0.05, ** *P*<0.01, *** *P*<0.001. **(D)** Infection does not modify ZO-1 mRNA expression. RT-PCR analysis of ZO-1 expression in response to *T.gondii* infection. Results were expressed as means ± SEM, n=4. The experiment was repeated twice independently with similar results.

Type I and especially type III interferons are known for playing a key role in upregulating or downregulating barrier permeability. For this reason, we studied the expression and localization of the tight junction scaffolding protein ZO-1 by confocal microscopy and RT-PCR in an outer blood-retina barrier (oBRB) model in response to interferon stimulation and *T. gondii* infection. As non-differentiated ARPE-19 cells do not form tight junctions, we first verified our differentiation protocol. Indeed, ARPE-19 cells differentiated during 8 weeks on transwell inserts in MEM-Nic medium showed strongly enhanced expression of primary RPE markers (**Figure S1A**), and still expressed IFNLR1 (**Figures S1G, H**). ZO-1 was present in non-differentiated RPE cells, but localized in vesicles throughout the cytoplasm, whereas it was localized at junctions in differentiated cells (**Figure S1E**). Interestingly, ZO-1 mRNA levels and electrical resistance were not impacted during differentiation (**Figures S1B, D**). Such differentiated RPE cells were then used as oBRB model. We first investigated the effect of interferon treatment on ZO-1 localization, by confocal microscopy. As shown in (**Figure 6A**), IFN-β, IFN-γ, and IFN-λ1 treatment increased ZO-1 tight junction localization, compared to PBS control. Image analysis confirmed this visual assessment, both for ZO-1 mean fluorescence intensity (MFI), as for total ZO-1 fluorescence, calculated by multiplying the ZO-1 MFI by the tight junction area (**Figure 6B**). This increase was more important with IFN-γ and IFN-λ1 than with IFN-β stimulation. A closer look at the junction structure also revealed slight changes in the tight junction morphology, such as increased occurrence of spikes and ruffles. (**Figures S2A–C**). Infection of cells with *T. gondii* RH strain resulted in localized increased ZO-1 expression but also gaps and broken areas (**Figure 6A**). Concomitant IFN-λ1 stimulation did not rescue the observed ‘infection’ phenotype. The inability of heat inactivated tachyzoites to induce any significant changes in ZO-1 localization and tight junction structure demonstrated that this effect was due to active parasite infection and/or virulence factor secretion. Interestingly, while image analysis revealed a significant increase in ZO-1 MFI upon infection, the total amount of fluorescence at the tight junction complex was not significantly different from the PBS control, due to the large gaps and broken areas (**Figure 6C**). This was in accordance with the absence of ZO-1 transcriptional activity in response to infection (**Figure 6D**). Infection thus seemed to induce a reorganization of ZO-1 protein within the junction rather than to modify its expression or trafficking to the junctions. To conclude, these results highlight the positive effect of interferon stimulation on ZO-1 tight junction localization and showed that *T. gondii* actively induces tight junction reorganization thus creating gaps and broken areas. Next, we wanted to see if this reorganization is associated with the permeability and barrier function of our BHRe model.

### IFN-β and IFN-λ1 increase barrier function in a STAT1-independent manner

**Figure 7:**
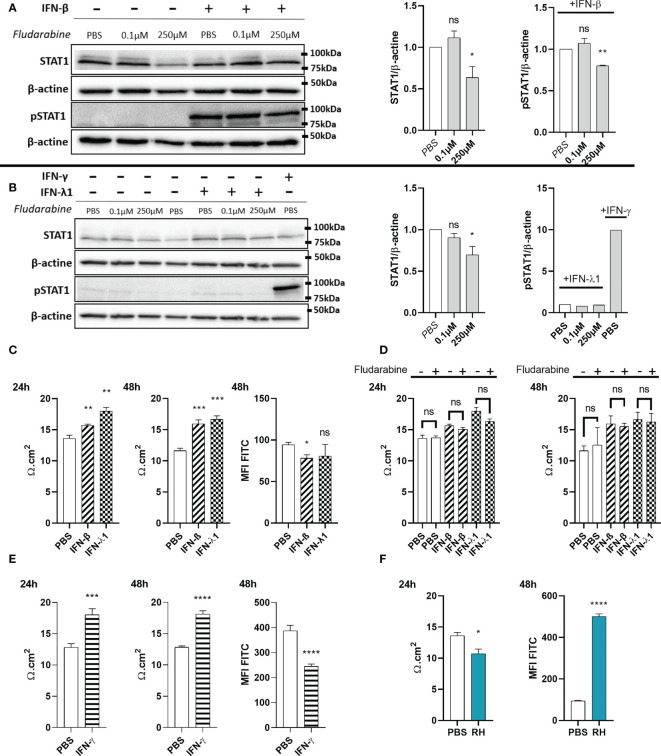
IFN-λ1 and IFN-β enhance barrier function in an STAT1 independent manner. **(A)** Western blots and their densitometric analysis of lysates from RPE cells treated with PBS, 0.1µM or 250µM fludarabine and stimulated with 20ng/mL IFN-β for 1 hours. Mean intensity of STAT1 and pSTAT1 were normalized to β-actin expression from the same sample. Results are expressed as means ± SEM of 2 independent experiments. **(B)** Western blots and their densitometric analysis of lysates from RPE cells treated with PBS, 0.1µM or 250µM fludarabine and stimulated with 20ng/mL IFN-λ1 for 3 hours or IFN-γ for 1 hour. Mean intensity of STAT1 and pSTAT1 were normalized to β-actin expression from the same sample. Results are expressed as means ± SEM of 2 independent experiments. **(C—F)** RPE cells differentiated for 8 weeks on transwell inserts were stimulated with 20ng/mL IFN-β, IFN-λ1 or IFN-γ and treated with 250µM fludarabine or infected with RH strain at a MOI of 1:1 as indicated. Electrical resistance was measured after 24h and 48h. FITC-dextran (1mg/mL) in culture medium was placed in the upper transwell chamber. Fluoresceine MFI in the lower chamber was measured 48h after 20ng/mL interferon stimulation or after 24h *T. gondii* RH strain infection. Results are expressed as means ± SEM, n=4. The experiment was repeated 3 times independently with similar results. ns: non significative, * *P*<0.05, ** *P*<0.01, *** *P*<0.001, **** *P*<0.0001, compared to PBS control unless otherwise indicated.

Since interferons caused increased ZO-1 junction localization in our oBRB model, we next studied their functional impact on permeability and tried to elucidate the underlying mechanisms. Particularly, studies of the role of STAT1 transduction gave contradictory results. ARPE-19 cells showed distinct STAT1 phosphorylation upon IFN-β stimulation (**Figure 7A**). To study the role of this activation, we treated the cells with the established STAT1 inhibitor fludarabine. We first verified the absence of detectable cell toxicity for all concentrations tested after a 72h treatment (**Figure S1C**). Then, we wanted to validate this inhibitor in our model. Fludarabine treatment resulted in a significant decrease of total STAT1 amount, as well as of STAT1 Tyr701 phosphorylation in response to IFN-β (**Figure 7A**). Regarding IFN-λ1, the amount of pSTAT1 Tyr701 was too low to be detected by Western blot (**Figure 7B**), possibly due to generally low expression of IFN-λ receptor (18), as was also evident for our model described in this study. Still, as IFN-β and IFN-λ1 use the same transduction pathways, we can assume that fludarabine also reduces STAT1 phosphorylation by IFN-λ1 stimulation.

To study the role of STAT1 and its activation, the oBRB model was stimulated with interferons, with or without fludarabine treatment (**Figures 7C, D**). Both IFN-β and IFN-λ1 significantly increased electrical barrier resistance at both 24h and 48h of stimulation. Fludarabine treatment did not modify electrical resistance in non-stimulated cells, as well as interferon stimulated cells, suggesting a STAT1 independent mechanism. Finally, barrier tightness was also studied in a 70kDa fluorescein-dextran permeability assay (**Figure 7C**). In accordance with the electrical resistance, IFN-β stimulation resulted in a significant decrease in FITC-dextran permeability. However, the decrease upon IFN-λ1 stimulation, did not reach statistical significance. We also tested the impact of IFN-γ treatment on electrical resistance and dextran permeability. Like type I and III interferon, IFN-γ also significantly enhanced electrical resistance and reduced dextran permeability (**Figure 7E**), in accordance with previously observed enhanced ZO-1 tight junction localization. Overall, these results show that type I and III, but also IFN-γ, are capable of enhancing ZO-1 tight junction localization, leading to reduced barrier permeability. This effect of type I and III interferons was independent of the canonical STAT1 transduction pathway.

### *Toxoplasma gondii* infection induces permeabilization of the outer blood-retina barrier

As our confocal microscopy images demonstrated tight junction reorganization and partial destruction by *T. gondii* infection, we also studied the impact of infection on barrier function. We observed a significant decrease in electrical resistance at 24h of infection, as well as a concordant strong increase of FITC-dextran permeability in infected cell cultures (**Figure 7F**). Microscopic examination showed the same aspect of an intact cell layer as in non-infected cells (not shown). These results demonstrate an active role of the parasite in diminishing barrier integrity and confirm that the reorganization observed by microscopy is linked to higher barrier permeability.

## Discussion

Here, we aimed to study the immune network regulating entry and installation of the parasite *T. gondii* in the retina. We specifically looked at the roles of type I and III interferons, known to regulate endothelial and epithelial barrier functions. For the blood-brain barrier, IFN-λ has been shown to limit virus passage (21). Unlike type II interferons, little is known about the involvement of type I and III interferons in the physiopathology of toxoplasmosis, particularly in the context of ocular toxoplasmosis (22). One of the central differences between these two types of interferons is the much more restricted expression of the receptor for IFN-λ. Our results show that all the cell lines in this study do express the IFN-λ receptor and are potentially activable by this cytokine, indicating that, in the eye, this receptor is not restricted to barrier cells. However, we found no significant effect of type I and III interferons on parasite proliferation in any cell type tested, in apparent contradiction to one study on IFN-β in ARPE-19 cells (23). This might be due to different experimental conditions. Generally, the effect of IFN-β on *T. gondii* proliferation has not been extensively investigated, and no study has yet looked at the effect of IFN-λ, so it is yet unknown if our results point to specific regulation mechanisms in cells within the CNS. The exact involvement of intracellular effector mechanisms employed by the different interferons in CNS and other cells could explain our results. Despite some redundancy in ISGs induced by each interferon they are considered to have mostly complementary roles in pathogen elimination (19). Some studies describe the promising role of ISG15, usually attributed to type I interferon stimulation, in autophagy-mediated parasite elimination (24). However, our results suggest this direct anti-parasitic mechanism to be insufficient in human ocular cell lines.

We then studied indirect effector mechanisms of the interferons in retinal cell types. We first observed that IFN-λ1 secretion was induced in all cell lines upon stimulation by IFN-β and IFN-γ. As both interferons, especially IFN-γ, are omnipresent during acute *T. gondii* infection, this means local presence of elevated levels of IFN-λ1 during infection. When comparing the effect of the interferons on the production of other cytokines, we see considerable overlap between IFN-β and IFN-γ, which induced an inflammatory secretion profile, also including chemokines attracting neutrophils and activated lymphocytes, confirming their role in immune activation. By contrast, IFN-λ1 induced much lower secretion of these inflammatory cytokines and does not seem to participate in this inflammatory loop. Interestingly, a recent study demonstrated even an active immunomodulatory role of IFN-λ1 (25). In light of our results, this cytokine could be used in the immune privileged ocular environment to enhance epithelial barrier functions without the inflammatory properties of the other interferons. This merits further studies, as it could functionally explain the observed abundant production of IFN-λ1. The basal secretion of this cytokine in RPE cells indicates a constant immunomodulatory role on other retinal cells. This lack of inflammatory activity of IFN-λ1, which we confirmed here for various retinal cell types, clearly distinguishes this cytokine from IFN-β, despite sharing a common STAT1-STAT2 heterodimer signaling pathway. This difference has been explained by absence of the more inflammatory STAT1 homodimers due to low density receptor expression (18), which we also describe here for the retinal cell lines.

Cellular reaction to *T. gondii* infection and the resulting clinical outcome of ocular toxoplasmosis are known to be highly strain-dependent (26). In our study, the expression of the inflammatory cytokines IL-6 and IL-8 was highly upregulated in response to infection with the virulent RH strain. This upregulation was particularly observed in synergy with interferon stimulation, demonstrating a dynamic interacting process of infection and interferon-induced cytokine production. In sharp contrast, no such upregulation was observed in Me49 infected cells, with some exception for IL-8. Even more, interferon induced IL-6 production was diminished in cells concomitantly infected with Me49. These opposite regulation patterns of inflammatory cytokines is consistent with the activation of STAT3 by virulent *T. gondii* strains, like RH, but not avirulent strains, such as Me49 (26). In interferon stimulated cells, secretion of chemokines CXCL10, CCL2 and CXCL9, known to be STAT1 dependent (27), was diminished by both parasite strains, and expression of Th1 cytokines, like IFN-β or IL-12, not observed. This indicates the action of the parasitic STAT1 reducing effector molecule TgIST (28) in our retinal cell lines.

Our previous human and mouse studies showed ocular expression of the signature cytokine IL-17A, which localized at least partially to Muller cells (13). The fact that the strong inflammatory response observed in our study did not include measurable levels of IL-17A could indicate that yet unknown factors by infiltrating cells are needed for IL-17A expression. Exploring the role of this cytokine, particularly in interaction with interferon stimulation, would be interesting, given its capacity to diminish barrier function (29). The barrier tightening action of IFN-λ is well described, especially for virus dissemination (30), but no study has looked at the retinal barrier. Our oBRB model of differentiated ARPE-19 cells allowed us to demonstrate the strong effect of different interferons on the intercellular localization of the tight junction protein ZO-1. In fact, IFN-β and more strongly IFN-γ and IFN-λ1 induced more abundant ZO-1 charged vesicles and increased ZO-1 positioning at the tight junction complex. The resultant structure of the tight junctions, with characteristic ruffles and spikes has been previously attributed to enhanced barrier efficiency (31). Remarkably, ARPE-19 cells are not capable of correctly expressing and positioning claudins at the tight junction complex (32). Claudins are the keystone of the pore pathway responsible for building a strong transepithelial electric resistance (TEER) (31). This leads to low electric resistance, also observed in other studies. The use of an established differentiation protocol (20) allowed us to approach primary RPE cell characteristics, like the expression of recognized markers and the ZO-1 localization to tight junctions. Even if this long differentiation step did not improve TEER, type I and III interferon stimulation of differentiated RPE cells did lead to increased electrical resistance and reduced dextran permeability. This suggests that other tight junction proteins than claudin can be recruited to tighten the barriers. Occludin is one of the candidates, as it is involved in the leak pathways allowing the passage of larger macromolecules (5). Even if the exact mechanisms remain to be elucidated, we showed that interferons are capable of outer blood retinal barrier tightening through ZO-1 reorganization at the cell membrane, an effect already described in other barrier models such as the blood-brain barrier (21, 33), intestinal barrier (34), and lung barrier (17). We confirmed here previous observations that *T. gondii* infection weakens ARPE-19 barrier function (35, 36), probably by secreting MMP like secreted excreted proteases (37), and showed the barrier preserving effect of interferon stimulation, even during concomitant infection.

The activation of the JAK/STAT pathway, demonstrated by abundant pTyr701 STAT1 in RPE cells following IFN-β stimulation is not surprising. The lack of detectable pTyrSTAT1 following IFN-λ1 stimulation, however, is likely due to much less efficient STAT1 stimulation by this cytokine, despite the signaling through the same signal pathway (19) and the limited sensitivity of Western blot. Modulation of ZO-1 localization and tight junction morphology was not affected by the STAT1 inhibitor fludarabine. Even if this treatment only partially diminished pSTAT1 band intensity, this difference should be enough to detect significant difference (38). Moreover, despite a much stronger STAT1 phosphorylation induced by IFN-β than IFN-λ1, we observed more efficient ZO-1 junction localization after IFN-λ1 treatment. Together, these results suggest a STAT1 independent mechanism of tight junction organization The same lack of STAT1 involvement was observed for tight junction formation at the blood-brain barrier (21), indicating a general link between interferon stimulation and tight junction regulation bypassing the canonical JAK/STAT pathway, by a yet unknown mechanism. Several non-canonical, STAT1 independent pathways have been described, such as the Crkl-C3G axis which activates RAP1 (39), known to participate in cell polarity and tight junction regulation in interaction with Shank2 (40).

In conclusion, we introduced an *in vitro* model to investigate the immune interaction between retinal cells, with particular focus on the role of type I and type III interferons. IFN-λ1 is readily produced by these cells by *T. gondii* infection and infection-induced cytokine responses. According to our results, these interferons do not act by controlling parasite proliferation, leaving this role to IFN-γ. Instead, they regulate the barrier function of the oBRB in a STAT1 independent manner. Further studies on how this influences passage of parasites, but also activated immune cells, into the retina are needed to assess the pathophysiological role of these interferons and possibly to identify immunological targets for this ocular disease.

## Data availability statement

The original contributions presented in the study are included in the article/**Supplementary Material**. Further inquiries can be directed to the corresponding author.

## Author contributions

BG: Conceptualization, Investigation, Methodology, Formal analysis, Software, Visualization, Data Writing – original draft, Writing – review and editing. VG: Conceptualization, Data curation, Formal analysis, Methodology, Investigation, Software, Writing – review and editing. CH: Investigation, Software, Writing – review and editing. CG and LB: Investigation, Writing – review and editing. JB and DF: Funding acquisitions, Resources, Writing – review and editing. OV and JD: Funding acquisition, Supervision, Writing – review and editing. AP: Project Administration, Supervision, Conceptualization, Investigation, Methodology, Formal analysis, Software, Visualization, Data Writing – original draft, Writing – review and editing. All authors contributed to the article and approved the submitted version.

## Supplementary material

Figure S1
**(A)** Differentiation protocol induces the expression of RPE primary cell markers. ARPE-19 cells were differentiated as explained in Material and Methods. The RT-qPCR results are shown as fold changes of cDNA copy numbers relative to non-differentiated ARPE-19 cells. N=1 with 3 independent experiments showing similar results. **(B)** Differentiation protocol does not modify ZO-1 expression. Quantification of ZO-1 mRNA by RT-PCR. RPE cells were differentiated in DMEM/F12 or MEM-Nic medium, as detailed in Material and Methods. Results are expressed as means ± SEM, n=4 with 2 independent experiments showing similar results. **(C)** Fludarabine does not affect differentiated ARPE-19 cell viability. After 8 weeks in MEM-Nic medium, cells were treated with 250µM fludarabine for 72h. Cell viability in supernatant and on adherent cells were assayed using trypan blue. Results are expressed as percentage ± SEM, n=4 with 3 independent experiments. **(D)** Differentiation protocol does not modify TEER in ARPE-19 cells. ARPE-19 cell were cultivated in DMEM/F-12 medium until reaching confluency or 8 weeks in MEM-Nicotinamide medium on laminin coated transwells (n=8). **(E)** Differentiation protocol is essential for correct ZO-1 junction localization. ARPE-19 cell were cultivated in DMEM/F-12 medium until reaching confluency (Non-diff.) or differentiated (Diff.) as detailed in Material and Methods. Cells were marked with anti ZO-1 (red) antibody and Hoechst33342 (blue). **(F)** Experimental procedure for BHRe treatments. After differentiation as detailed in Material and Methods, RPE cells were infected by introducing parasites in the upper transwell chamber. For interferon stimulation or fludarabine treatment, the medium of the upper and lower chambers was replaced with fresh medium containing 20ng/mL interferon or 250µM fludarabine. **(G)** Differentiated ARPE-19 cells express the IFNLR1 receptor subunit (Flow cytometry). Differentiated cells were prepared for flow cytometry analysis as described in material and methods. The MFI of IFNLR1 marked cells (blue) were compared with IgG isotype marked control (red). **(H)** Differentiated ARPE-19 cells express the IFNLR1 receptor subunit (Microscopy). Confocal microscopy image of DMEM/F-12 cultivated (Non-diff.) or differentiated (Diff.) RPE cells. The cells were fixed, permeabilized and the IFNLR1 subunit and cell nuclei labeled with AlexaFluor488 (green) and Hoechst 33342 (blue), respectively.

Figure S2 Interferon stimulation induces tight junction morphology changes. Differentiated RPE cells were stimulated with interferons or PBS (as indicated on panels) and marked with anti-ZO1-Alexa555 (red) antibody and Hoechst33342 (blue). Z-stack sections were made using confocal microscopy. 3D images with surface rendering were then created with Imaris. **(A)** Two different views of the same cell were represented and tight junction ruffles are indicated by arrows. **(B)** Top views of 3D images with surface rendering on PBS control and IFN-λ1 treated cells. **(C)** IFN-λ1 enhances the amount of cytoplasmic vesicles containing ZO-1 proteins (side view).

Table S1 FIJI macro used for sample analysis.

Table S2 Raw experiment data.

## References

[1] Forrester JV McMenamin PG Dando SJ. CNS infection and immune privilege. Nat Rev Neurosci (2018) 19(11):655−71. doi: 10.1038/s41583-018-0070-8 30310148

[2] Weidner JM Kanatani S Hernández-Castañeda MA Fuks JM Rethi B Wallin RPA. Rapid cytoskeleton remodelling in dendritic cells following invasion by toxoplasma gondii coincides with the onset of a hypermigratory phenotype. Cell Microbiol (2013) 15(10):1735−52. doi: 10.1111/cmi.12145 23534541

[3] Lambert H Barragan A. Modelling parasite dissemination: host cell subversion and immune evasion by toxoplasma gondii. Cell Microbiol (2010) 12(3):292−300. doi: 10.1111/j.1462-5822.2009.01417.x 19995386

[4] O’Leary F Campbell M. The blood-retina barrier in health and disease. FEBS J (2021) 290(4):878–91. doi: 10.1111/febs.16330 34923749

[5] Otani T Furuse M. Tight junction structure and function revisited. Trends Cell Biol (2020) 30(10):805−17. doi: 10.1016/j.tcb.2020.08.004 32891490

[6] Umeda K Ikenouchi J Katahira-Tayama S Furuse K Sasaki H Nakayama M. ZO-1 and ZO-2 independently determine where claudins are polymerized in tight-junction strand formation. Cell (2006) 126(4):741−54. doi: 10.1016/j.cell.2006.06.043 16923393

[7] Ivashkiv LB Donlin LT. Regulation of type I interferon responses. Nat Rev Immunol (2014) 14(1):36−49. doi: 10.1038/nri3581 24362405 PMC4084561

[8] Schroder K Hertzog PJ Ravasi T Hume DA. Interferon-gamma: an overview of signals, mechanisms and functions. J Leukoc Biol (2004) 75(2):163−89. doi: 10.1189/jlb.0603252 14525967

[9] Ank N West H Paludan SR. IFN-lambda: novel antiviral cytokines. J Interferon Cytokine Res (2006) 26(6):373−9. doi: 10.1089/jir.2006.26.373 16734557

[10] Murray PJ. The JAK-STAT signaling pathway: input and output integration. J Immunol (2007) 178(5):2623−9. doi: 10.4049/jimmunol.178.5.2623 17312100

[11] Green R Ireton RC Gale M. Interferon-stimulated genes: new platforms and computational approaches. Mamm Genome (2018) 29(7−8):593−602. doi: 10.1007/s00335-018-9755-6 29982912

[12] Rochet E Brunet J Sabou M Marcellin L Bourcier T Candolfi E. Interleukin-6-driven inflammatory response induces retinal pathology in a model of ocular toxoplasmosis reactivation. Infect Immun (2015) 83(5):2109−17. doi: 10.1128/IAI.02985-14 25754200 PMC4399048

[13] Sauer A Pfaff AW Villard O Creuzot-Garcher C Dalle F Chiquet C. Interleukin 17A as an effective target for anti-inflammatory and antiparasitic treatment of toxoplasmic uveitis. J Infect Dis (2012) 206(8):1319−29. doi: 10.1093/infdis/jis486 22927448

[14] Sturge CR Yarovinsky F. Complex immune cell interplay in the gamma interferon response during toxoplasma gondii infection. Infect Immun (2014) 82(8):3090−7. doi: 10.1128/IAI.01722-14 24866795 PMC4136216

[15] Mahmoud ME Ui F Salman D Nishimura M Nishikawa Y. Mechanisms of interferon-beta-induced inhibition of toxoplasma gondii growth in murine macrophages and embryonic fibroblasts: role of immunity-related GTPase M1. Cell Microbiol (2015) 17(7):1069−83. doi: 10.1111/cmi.12423 25628099

[16] Lazear HM Nice TJ Diamond MS. Interferon-λ: immune functions at barrier surfaces and beyond. Immunity (2015) 43(1):15−28. doi: 10.1016/j.immuni.2015.07.001 26200010 PMC4527169

[17] Ye L Schnepf D Staeheli P. Interferon-λ orchestrates innate and adaptive mucosal immune responses. Nat Rev Immunol (2019) 19(10):614−25. doi: 10.1038/s41577-019-0182-z 31201377

[18] Forero A Ozarkar S Li H Lee CH Hemann EA Nadjsombati MS. Differential activation of the transcription factor IRF1 underlies the distinct immune responses elicited by type I and type III interferons. Immunity (2019). 51 (3):451–64.e6. doi: 10.1016/j.immuni.2019.07.007 PMC7447158 31471108

[19] Lazear HM Schoggins JW Diamond MS. Shared and distinct functions of type I and type III interferons. Immunity (2019) 50(4):907−23. doi: 10.1016/j.immuni.2019.03.025 30995506 PMC6839410

[20] Hazim RA Volland S Yen A Burgess BL Williams DS. Rapid differentiation of the human RPE cell line, ARPE-19, induced by nicotinamide. Exp Eye Res (2019) 179:18−24. doi: 10.1016/j.exer.2018.10.009 30336127 PMC6360117

[21] Lazear HM Daniels BP Pinto AK Huang AC Vick SC Doyle SE. Interferon-λ restricts West Nile virus neuroinvasion by tightening the blood-brain barrier. Sci Transl Med (2015) 7(284):284ra59. doi: 10.1126/scitranslmed.aaa4304 PMC4435724 25904743

[22] Greigert V Bittich-Fahmi F Pfaff AW. Pathophysiology of ocular toxoplasmosis: facts and open questions. PloS Negl Trop Dis (2020) 14(12):e0008905. doi: 10.1371/journal.pntd.0008905 33382688 PMC7774838

[23] Nagineni CN Pardhasaradhi K Martins MC Detrick B Hooks JJ. Mechanisms of interferon-induced inhibition of toxoplasma gondii replication in human retinal pigment epithelial cells. Infect Immun (1996) 64(10):4188−96. doi: 10.1128/iai.64.10.4188-4196.1996 8926087 PMC174355

[24] Bhushan J Radke JB Perng YC Mcallaster M Lenschow DJ Virgin HW. ISG15 connects autophagy and IFN-γ-Dependent control of toxoplasma gondii infection in human cells. mBio (2020) 11(5):e00852–20. doi: 10.1128/mBio.00852-20 PMC7542356 33024031

[25] Zanoni I Granucci F Broggi A. Interferon (IFN)-λ takes the helm: immunomodulatory roles of type III IFNs. Front Immunol (2017) 8:1661. doi: 10.3389/fimmu.2017.01661 29234323 PMC5712353

[26] Melo MB Jensen KD Saeij JP. Toxoplasma gondii effectors are master regulators of the inflammatory response. Trends Parasitol (2011) 27(11):487–95. doi: 10.1016/j.pt.2011.08.001 PMC3200456 21893432

[27] Casazza RL Lazear HM. Why is IFN-λ less inflammatory? one IRF decides. Immunity (2019) 51(3):415−7. doi: 10.1016/j.immuni.2019.08.019 31533051

[28] Gay G Braun L Brenier-Pinchart MP Vollaire J Josserand V Bertini RL. Toxoplasma gondii TgIST co-opts host chromatin repressors dampening STAT1-dependent gene regulation and IFN-gamma-mediated host defenses. J Exp Med (2016) 213(9):1779−98. doi: 10.1084/jem.20160340 27503074 PMC4995087

[29] Chen Y Yang P Li F Kijlstra A. The effects of Th17 cytokines on the inflammatory mediator production and barrier function of ARPE-19 cells. PloS One (2011) 6(3):e18139. doi: 10.1371/journal.pone.0018139 21479174 PMC3068165

[30] Wells AI Coyne CB. Type III interferons in antiviral defenses at barrier surfaces. Trends Immunol (2018) 39(10):848−58. doi: 10.1016/j.it.2018.08.008 30219309 PMC6179363

[31] Lynn KS Peterson RJ Koval M. Ruffles and spikes: control of tight junction morphology and permeability by claudins. Biochim Biophys Acta Biomembr (2020) 1862(9):183339. doi: 10.1016/j.bbamem.2020.183339 32389670 PMC7299829

[32] Peng S Wang SB Singh D Zhao PYC Davis K Chen B. Claudin-3 and claudin-19 partially restore native phenotype to ARPE-19 cells via effects on tight junctions and gene expression. Exp Eye Res (2016) 151:179−89. doi: 10.1016/j.exer.2016.08.021 27593915

[33] Douam F Soto Albrecht YE Hrebikova G Sadimin E Davidson C Kotenko SV. Type III interferon-mediated signaling is critical for controlling live attenuated yellow fever virus infection *In Vivo* . mBio (2022) 8(4):e00819–17. doi: 10.1128/mBio.00819-17 PMC5559630 28811340

[34] Odendall C Voak AA Kagan JC. Type III IFNs are commonly induced by bacteria-sensing TLRs and reinforce epithelial barriers during infection. J Immunol (2017) 199(9):3270−9. doi: 10.4049/jimmunol.1700250 28954888 PMC5679450

[35] Nogueira AR Leve F Morgado-Diaz J Tedesco RC Pereira MCS. Effect of toxoplasma gondii infection on the junctional complex of retinal pigment epithelial cells. Parasitology (2016) 143(5):568−75. doi: 10.1017/S0031182015001973 26928468

[36] Song HB Jun HO Kim JH Lee YH Choi MH Kim JH. Disruption of outer blood-retinal barrier by toxoplasma gondii-infected monocytes is mediated by paracrinely activated FAK signaling. PloS One (2017) 12(4):e0175159. doi: 10.1371/journal.pone.0175159 28406972 PMC5390985

[37] Rosenberg A Sibley LD. Toxoplasma gondii secreted effectors co-opt host repressor complexes to inhibit necroptosis. Cell Host Microbe (2021) 29(7):1186–1198.e8. doi: 10.1016/j.chom.2021.04.016 34043960 PMC8711274

[38] Frank DA Mahajan S Ritz J. Fludarabine-induced immunosuppression is associated with inhibition of STAT1 signaling. Nat Med (1999) 5(4):444−7. doi: 10.1038/7445 10202937

[39] Mazewski C Perez RE Fish EN Platanias LC. Type I interferon (IFN)-regulated activation of canonical and non-canonical signaling pathways. Front Immunol (2020) 11:606456. doi: 10.3389/fimmu.2020.606456 33329603 PMC7719805

[40] Sasaki K Kojitani N Hirose H Yoshihama Y Suzuki H Shimada M. Shank2 binds to aPKC and controls tight junction formation with Rap1 signaling during establishment of epithelial cell polarity. Cell Rep avr (2020) 31(1):107407. doi: 10.1016/j.celrep.2020.02.088 32268103

