# Supplementary figures and images for: Type I and III interferons shape the retinal cytokine network and barrier function in an in vitro model of ocular toxoplasmosis
Source: Front Immunol. 2023 May 2;14:1148037. doi: 10.3389/fimmu.2023.1148037 (PMC10188120; doi:10.3389/fimmu.2023.1148037)

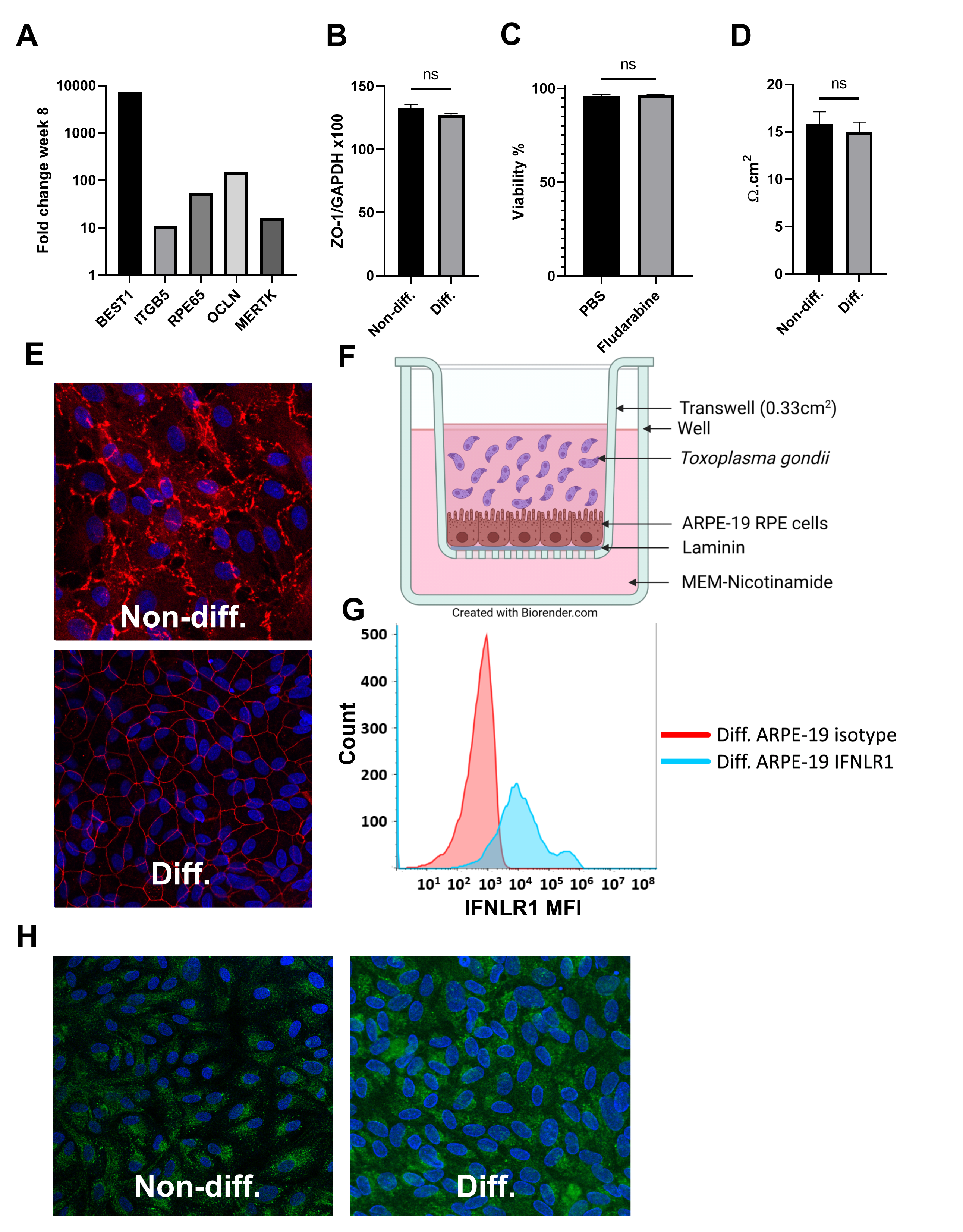

Supplement: Figure S1 — (A) Differentiation protocol induces the expression of RPE primary cell markers. ARPE-19 cells were differentiated as explained in Material and Methods. The RT-qPCR results are shown as fold changes of cDNA copy numbers relative to non-differentiated ARPE-19 cells. N=1 with 3 independent experiments showing similar results. (B) Differentiation protocol does not modify ZO-1 expression. Quantification of ZO-1 mRNA by RT-PCR. RPE cells were differentiated in DMEM/F12 or MEM-Nic medium, as detailed in Material and Methods. Results are expressed as means ± SEM, n=4 with 2 independent experiments showing similar results. (C) Fludarabine does not affect differentiated ARPE-19 cell viability. After 8 weeks in MEM-Nic medium, cells were treated with 250µM fludarabine for 72h. Cell viability in supernatant and on adherent cells were assayed using trypan blue. Results are expressed as percentage ± SEM, n=4 with 3 independent experiments. (D) Differentiation protocol does not modify TEER in ARPE-19 cells. ARPE-19 cell were cultivated in DMEM/F-12 medium until reaching confluency or 8 weeks in MEM-Nicotinamide medium on laminin coated transwells (n=8). (E) Differentiation protocol is essential for correct ZO-1 junction localization. ARPE-19 cell were cultivated in DMEM/F-12 medium until reaching confluency (Non-diff.) or differentiated (Diff.) as detailed in Material and Methods. Cells were marked with anti ZO-1 (red) antibody and Hoechst33342 (blue). (F) Experimental procedure for BHRe treatments. After differentiation as detailed in Material and Methods, RPE cells were infected by introducing parasites in the upper transwell chamber. For interferon stimulation or fludarabine treatment, the medium of the upper and lower chambers was replaced with fresh medium containing 20ng/mL interferon or 250µM fludarabine. (G) Differentiated ARPE-19 cells express the IFNLR1 receptor subunit (Flow cytometry). Differentiated cells were prepared for flow cytometry analysis as described in mate [file Image_1.tif]

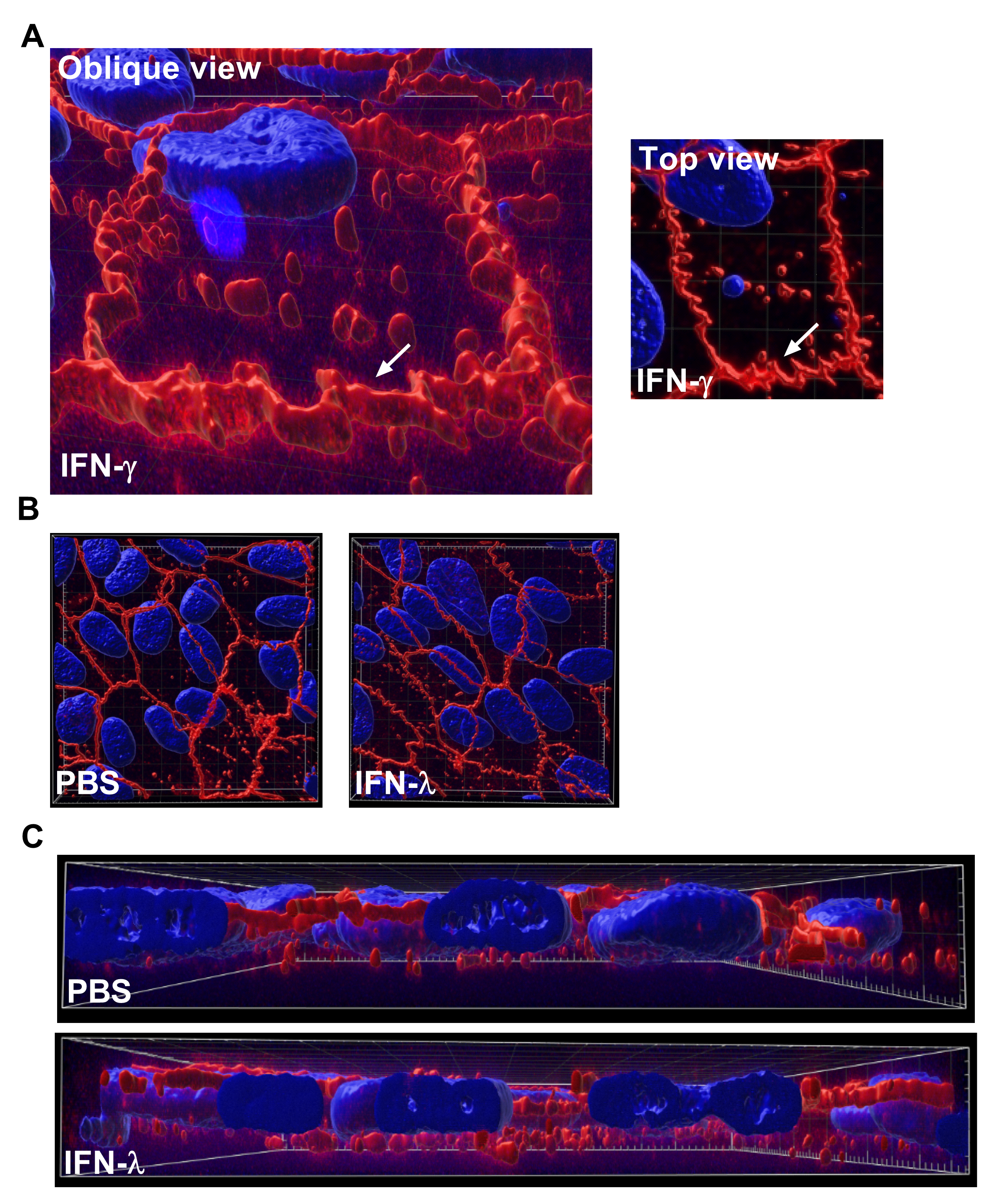

Supplement: Figure S2 — Interferon stimulation induces tight junction morphology changes. Differentiated RPE cells were stimulated with interferons or PBS (as indicated on panels) and marked with anti-ZO1-Alexa555 (red) antibody and Hoechst33342 (blue). Z-stack sections were made using confocal microscopy. 3D images with surface rendering were then created with Imaris. (A) Two different views of the same cell were represented and tight junction ruffles are indicated by arrows. (B) Top views of 3D images with surface rendering on PBS control and IFN-λ1 treated cells. (C) IFN-λ1 enhances the amount of cytoplasmic vesicles containing ZO-1 proteins (side view). [file Image_2.tif]
