# Supplementary material for: Type I and III interferons shape the retinal cytokine network and barrier function in an in vitro model of ocular toxoplasmosis
Source: Front Immunol. 2023 May 2;14:1148037. doi: 10.3389/fimmu.2023.1148037 (PMC10188120; doi:10.3389/fimmu.2023.1148037)
Supplement: Table S1 — FIJI macro used for sample analysis. [file Table_1.docx]

open("C:/Users/Data source/c1-1.czi");

run("Duplicate...", " ");

run("Subtract Background...", "rolling=10 sliding");

run("Auto Threshold", "method=Default white");

run("Convert to Mask");

run("Erode");

run("Create Selection");

selectWindow("C:/Users/Data source/c1-1.czi - C=1");

run("Subtract Background...", "rolling=10 sliding");

run("Restore Selection");

run("Measure");

selectWindow("C:/Users/Data source/c1-1.czi - C=1");

close();

selectWindow("C:/Users/Data source/c1-1.czi - C=0");

close();

selectWindow("C:/Users/Data source/c1-1.czi - C=1-1");

close();

open("C:/Users/Data source/c1-2.czi");

run("Duplicate...", " ");

run("Subtract Background...", "rolling=10 sliding");

run("Auto Threshold", "method=Default white");

run("Convert to Mask");

run("Erode");

run("Create Selection");

selectWindow("C:/Users/Data source/c1-2.czi - C=1");

run("Subtract Background...", "rolling=10 sliding");

run("Restore Selection");

run("Measure");

selectWindow("C:/Users/Data source/c1-2.czi - C=1");

close();

selectWindow("C:/Users/Data source/c1-2.czi - C=0");

close();

selectWindow("C:/Users/Data source/c1-2.czi - C=1-1");

close();

open("C:/Users/Data source/c1-3.czi");

run("Duplicate...", " ");

run("Subtract Background...", "rolling=10 sliding");

run("Auto Threshold", "method=Default white");

run("Convert to Mask");

run("Erode");

run("Create Selection");

selectWindow("C:/Users/Data source/c1-3.czi - C=1");

run("Subtract Background...", "rolling=10 sliding");

run("Restore Selection");

run("Measure");

selectWindow("C:/Users/Data source/c1-3.czi - C=1");

close();

selectWindow("C:/Users/Data source/c1-3.czi - C=0");

close();

selectWindow("C:/Users/Data source/c1-3.czi - C=1-1");

close();

open("C:/Users/Data source/c2-1.czi");

run("Duplicate...", " ");

run("Subtract Background...", "rolling=10 sliding");

run("Auto Threshold", "method=Default white");

run("Convert to Mask");

run("Erode");

run("Create Selection");

selectWindow("C:/Users/Data source/c2-1.czi - C=1");

run("Subtract Background...", "rolling=10 sliding");

run("Restore Selection");

run("Measure");

selectWindow("C:/Users/Data source/c2-1.czi - C=1");

close();

selectWindow("C:/Users/Data source/c2-1.czi - C=0");

close();

selectWindow("C:/Users/Data source/c2-1.czi - C=1-1");

close();

open("C:/Users/Data source/c2-2.czi");

run("Duplicate...", " ");

run("Subtract Background...", "rolling=10 sliding");

run("Auto Threshold", "method=Default white");

run("Convert to Mask");

run("Erode");

run("Create Selection");

selectWindow("C:/Users/Data source/c2-2.czi - C=1");

run("Subtract Background...", "rolling=10 sliding");

run("Restore Selection");

run("Measure");

selectWindow("C:/Users/Data source/c2-2.czi - C=1");

close();

selectWindow("C:/Users/Data source/c2-2.czi - C=0");

close();

selectWindow("C:/Users/Data source/c2-2.czi - C=1-1");

close();

open("C:/Users/Data source/c2-3.czi");

run("Duplicate...", " ");

run("Subtract Background...", "rolling=10 sliding");

run("Auto Threshold", "method=Default white");

run("Convert to Mask");

run("Erode");

run("Create Selection");

selectWindow("C:/Users/Data source/c2-3.czi - C=1");

run("Subtract Background...", "rolling=10 sliding");

run("Restore Selection");

run("Measure");

selectWindow("C:/Users/Data source/c2-3.czi - C=1");

close();

selectWindow("C:/Users/Data source/c2-3.czi - C=0");

close();

selectWindow("C:/Users/Data source/c2-3.czi - C=1-1");

close();

open("C:/Users/Data source/c3-1.czi");

run("Duplicate...", " ");

run("Subtract Background...", "rolling=10 sliding");

run("Auto Threshold", "method=Default white");

run("Convert to Mask");

run("Erode");

run("Create Selection");

selectWindow("C:/Users/Data source/c3-1.czi - C=1");

run("Subtract Background...", "rolling=10 sliding");

run("Restore Selection");

run("Measure");

selectWindow("C:/Users/Data source/c3-1.czi - C=1");

close();

selectWindow("C:/Users/Data source/c3-1.czi - C=0");

close();

selectWindow("C:/Users/Data source/c3-1.czi - C=1-1");

close();

open("C:/Users/Data source/c3-2.czi");

run("Duplicate...", " ");

run("Subtract Background...", "rolling=10 sliding");

run("Auto Threshold", "method=Default white");

run("Convert to Mask");

run("Erode");

run("Create Selection");

selectWindow("C:/Users/Data source/c3-2.czi - C=1");

run("Subtract Background...", "rolling=10 sliding");

run("Restore Selection");

run("Measure");

selectWindow("C:/Users/Data source/c3-2.czi - C=1");

close();

selectWindow("C:/Users/Data source/c3-2.czi - C=0");

close();

selectWindow("C:/Users/Data source/c3-2.czi - C=1-1");

close();

open("C:/Users/Data source/c3-3.czi");

run("Duplicate...", " ");

run("Subtract Background...", "rolling=10 sliding");

run("Auto Threshold", "method=Default white");

run("Convert to Mask");

run("Erode");

run("Create Selection");

selectWindow("C:/Users/Data source/c3-3.czi - C=1");

run("Subtract Background...", "rolling=10 sliding");

run("Restore Selection");

run("Measure");

selectWindow("C:/Users/Data source/c3-3.czi - C=1");

close();

selectWindow("C:/Users/Data source/c3-3.czi - C=0");

close();

selectWindow("C:/Users/Data source/c3-3.czi - C=1-1");

close();

open("C:/Users/Data source/l1-1.czi");

run("Duplicate...", " ");

run("Subtract Background...", "rolling=10 sliding");

run("Auto Threshold", "method=Default white");

run("Convert to Mask");

run("Erode");

run("Create Selection");

selectWindow("C:/Users/Data source/l1-1.czi - C=1");

run("Subtract Background...", "rolling=10 sliding");

run("Restore Selection");

run("Measure");

selectWindow("C:/Users/Data source/l1-1.czi - C=1");

close();

selectWindow("C:/Users/Data source/l1-1.czi - C=0");

close();

selectWindow("C:/Users/Data source/l1-1.czi - C=1-1");

close();

open("C:/Users/Data source/l1-2.czi");

run("Duplicate...", " ");

run("Subtract Background...", "rolling=10 sliding");

run("Auto Threshold", "method=Default white");

run("Convert to Mask");

run("Erode");

run("Create Selection");

selectWindow("C:/Users/Data source/l1-2.czi - C=1");

run("Subtract Background...", "rolling=10 sliding");

run("Restore Selection");

run("Measure");

selectWindow("C:/Users/Data source/l1-2.czi - C=1");

close();

selectWindow("C:/Users/Data source/l1-2.czi - C=0");

close();

selectWindow("C:/Users/Data source/l1-2.czi - C=1-1");

close();

open("C:/Users/Data source/l1-3.czi");

run("Duplicate...", " ");

run("Subtract Background...", "rolling=10 sliding");

run("Auto Threshold", "method=Default white");

run("Convert to Mask");

run("Erode");

run("Create Selection");

selectWindow("C:/Users/Data source/l1-3.czi - C=1");

run("Subtract Background...", "rolling=10 sliding");

run("Restore Selection");

run("Measure");

selectWindow("C:/Users/Data source/l1-3.czi - C=1");

close();

selectWindow("C:/Users/Data source/l1-3.czi - C=0");

close();

selectWindow("C:/Users/Data source/l1-3.czi - C=1-1");

close();

open("C:/Users/Data source/l2-1.czi");

run("Duplicate...", " ");

run("Subtract Background...", "rolling=10 sliding");

run("Auto Threshold", "method=Default white");

run("Convert to Mask");

run("Erode");

run("Create Selection");

selectWindow("C:/Users/Data source/l2-1.czi - C=1");

run("Subtract Background...", "rolling=10 sliding");

run("Restore Selection");

run("Measure");

selectWindow("C:/Users/Data source/l2-1.czi - C=1");

close();

selectWindow("C:/Users/Data source/l2-1.czi - C=0");

close();

selectWindow("C:/Users/Data source/l2-1.czi - C=1-1");

close();

open("C:/Users/Data source/l2-2.czi");

run("Duplicate...", " ");

run("Subtract Background...", "rolling=10 sliding");

run("Auto Threshold", "method=Default white");

run("Convert to Mask");

run("Erode");

run("Create Selection");

selectWindow("C:/Users/Data source/l2-2.czi - C=1");

run("Subtract Background...", "rolling=10 sliding");

run("Restore Selection");

run("Measure");

selectWindow("C:/Users/Data source/l2-2.czi - C=1");

close();

selectWindow("C:/Users/Data source/l2-2.czi - C=0");

close();

selectWindow("C:/Users/Data source/l2-2.czi - C=1-1");

close();

open("C:/Users/Data source/l2-3.czi");

run("Duplicate...", " ");

run("Subtract Background...", "rolling=10 sliding");

run("Auto Threshold", "method=Default white");

run("Convert to Mask");

run("Erode");

run("Create Selection");

selectWindow("C:/Users/Data source/l2-3.czi - C=1");

run("Subtract Background...", "rolling=10 sliding");

run("Restore Selection");

run("Measure");

selectWindow("C:/Users/Data source/l2-3.czi - C=1");

close();

selectWindow("C:/Users/Data source/l2-3.czi - C=0");

close();

selectWindow("C:/Users/Data source/l2-3.czi - C=1-1");

close();

open("C:/Users/Data source/l2-4.czi");

run("Duplicate...", " ");

run("Subtract Background...", "rolling=10 sliding");

run("Auto Threshold", "method=Default white");

run("Convert to Mask");

run("Erode");

run("Create Selection");

selectWindow("C:/Users/Data source/l2-4.czi - C=1");

run("Subtract Background...", "rolling=10 sliding");

run("Restore Selection");

run("Measure");

selectWindow("C:/Users/Data source/l2-4.czi - C=1");

close();

selectWindow("C:/Users/Data source/l2-4.czi - C=0");

close();

selectWindow("C:/Users/Data source/l2-4.czi - C=1-1");

close();

open("C:/Users/Data source/l2-5.czi");

run("Duplicate...", " ");

run("Subtract Background...", "rolling=10 sliding");

run("Auto Threshold", "method=Default white");

run("Convert to Mask");

run("Erode");

run("Create Selection");

selectWindow("C:/Users/Data source/l2-5.czi - C=1");

run("Subtract Background...", "rolling=10 sliding");

run("Restore Selection");

run("Measure");

selectWindow("C:/Users/Data source/l2-5.czi - C=1");

close();

selectWindow("C:/Users/Data source/l2-5.czi - C=0");

close();

selectWindow("C:/Users/Data source/l2-5.czi - C=1-1");

close();

open("C:/Users/Data source/l3-1.czi");

run("Duplicate...", " ");

run("Subtract Background...", "rolling=10 sliding");

run("Auto Threshold", "method=Default white");

run("Convert to Mask");

run("Erode");

run("Create Selection");

selectWindow("C:/Users/Data source/l3-1.czi - C=1");

run("Subtract Background...", "rolling=10 sliding");

run("Restore Selection");

run("Measure");

selectWindow("C:/Users/Data source/l3-1.czi - C=1");

close();

selectWindow("C:/Users/Data source/l3-1.czi - C=0");

close();

selectWindow("C:/Users/Data source/l3-1.czi - C=1-1");

close();

open("C:/Users/Data source/l3-2.czi");

run("Duplicate...", " ");

run("Subtract Background...", "rolling=10 sliding");

run("Auto Threshold", "method=Default white");

run("Convert to Mask");

run("Erode");

run("Create Selection");

selectWindow("C:/Users/Data source/l3-2.czi - C=1");

run("Subtract Background...", "rolling=10 sliding");

run("Restore Selection");

run("Measure");

selectWindow("C:/Users/Data source/l3-2.czi - C=1");

close();

selectWindow("C:/Users/Data source/l3-2.czi - C=0");

close();

selectWindow("C:/Users/Data source/l3-2.czi - C=1-1");

close();

open("C:/Users/Data source/l3-3.czi");

run("Duplicate...", " ");

run("Subtract Background...", "rolling=10 sliding");

run("Auto Threshold", "method=Default white");

run("Convert to Mask");

run("Erode");

run("Create Selection");

selectWindow("C:/Users/Data source/l3-3.czi - C=1");

run("Subtract Background...", "rolling=10 sliding");

run("Restore Selection");

run("Measure");

selectWindow("C:/Users/Data source/l3-3.czi - C=1");

close();

selectWindow("C:/Users/Data source/l3-3.czi - C=0");

close();

selectWindow("C:/Users/Data source/l3-3.czi - C=1-1");

close();

open("C:/Users/Data source/b1-1.czi");

run("Duplicate...", " ");

run("Subtract Background...", "rolling=10 sliding");

run("Auto Threshold", "method=Default white");

run("Convert to Mask");

run("Erode");

run("Create Selection");

selectWindow("C:/Users/Data source/b1-1.czi - C=1");

run("Subtract Background...", "rolling=10 sliding");

run("Restore Selection");

run("Measure");

selectWindow("C:/Users/Data source/b1-1.czi - C=1");

close();

selectWindow("C:/Users/Data source/b1-1.czi - C=0");

close();

selectWindow("C:/Users/Data source/b1-1.czi - C=1-1");

close();

open("C:/Users/Data source/b1-2.czi");

run("Duplicate...", " ");

run("Subtract Background...", "rolling=10 sliding");

run("Auto Threshold", "method=Default white");

run("Convert to Mask");

run("Erode");

run("Create Selection");

selectWindow("C:/Users/Data source/b1-2.czi - C=1");

run("Subtract Background...", "rolling=10 sliding");

run("Restore Selection");

run("Measure");

selectWindow("C:/Users/Data source/b1-2.czi - C=1");

close();

selectWindow("C:/Users/Data source/b1-2.czi - C=0");

close();

selectWindow("C:/Users/Data source/b1-2.czi - C=1-1");

close();

open("C:/Users/Data source/b1-3.czi");

run("Duplicate...", " ");

run("Subtract Background...", "rolling=10 sliding");

run("Auto Threshold", "method=Default white");

run("Convert to Mask");

run("Erode");

run("Create Selection");

selectWindow("C:/Users/Data source/b1-3.czi - C=1");

run("Subtract Background...", "rolling=10 sliding");

run("Restore Selection");

run("Measure");

selectWindow("C:/Users/Data source/b1-3.czi - C=1");

close();

selectWindow("C:/Users/Data source/b1-3.czi - C=0");

close();

selectWindow("C:/Users/Data source/b1-3.czi - C=1-1");

close();

open("C:/Users/Data source/b2-1.czi");

run("Duplicate...", " ");

run("Subtract Background...", "rolling=10 sliding");

run("Auto Threshold", "method=Default white");

run("Convert to Mask");

run("Erode");

run("Create Selection");

selectWindow("C:/Users/Data source/b2-1.czi - C=1");

run("Subtract Background...", "rolling=10 sliding");

run("Restore Selection");

run("Measure");

selectWindow("C:/Users/Data source/b2-1.czi - C=1");

close();

selectWindow("C:/Users/Data source/b2-1.czi - C=0");

close();

selectWindow("C:/Users/Data source/b2-1.czi - C=1-1");

close();

open("C:/Users/Data source/b2-2.czi");

run("Duplicate...", " ");

run("Subtract Background...", "rolling=10 sliding");

run("Auto Threshold", "method=Default white");

run("Convert to Mask");

run("Erode");

run("Create Selection");

selectWindow("C:/Users/Data source/b2-2.czi - C=1");

run("Subtract Background...", "rolling=10 sliding");

run("Restore Selection");

run("Measure");

selectWindow("C:/Users/Data source/b2-2.czi - C=1");

close();

selectWindow("C:/Users/Data source/b2-2.czi - C=0");

close();

selectWindow("C:/Users/Data source/b2-2.czi - C=1-1");

close();

open("C:/Users/Data source/b2-3.czi");

run("Duplicate...", " ");

run("Subtract Background...", "rolling=10 sliding");

run("Auto Threshold", "method=Default white");

run("Convert to Mask");

run("Erode");

run("Create Selection");

selectWindow("C:/Users/Data source/b2-3.czi - C=1");

run("Subtract Background...", "rolling=10 sliding");

run("Restore Selection");

run("Measure");

selectWindow("C:/Users/Data source/b2-3.czi - C=1");

close();

selectWindow("C:/Users/Data source/b2-3.czi - C=0");

close();

selectWindow("C:/Users/Data source/b2-3.czi - C=1-1");

close();

open("C:/Users/Data source/b3-1.czi");

run("Duplicate...", " ");

run("Subtract Background...", "rolling=10 sliding");

run("Auto Threshold", "method=Default white");

run("Convert to Mask");

run("Erode");

run("Create Selection");

selectWindow("C:/Users/Data source/b3-1.czi - C=1");

run("Subtract Background...", "rolling=10 sliding");

run("Restore Selection");

run("Measure");

selectWindow("C:/Users/Data source/b3-1.czi - C=1");

close();

selectWindow("C:/Users/Data source/b3-1.czi - C=0");

close();

selectWindow("C:/Users/Data source/b3-1.czi - C=1-1");

close();

open("C:/Users/Data source/b3-2.czi");

run("Duplicate...", " ");

run("Subtract Background...", "rolling=10 sliding");

run("Auto Threshold", "method=Default white");

run("Convert to Mask");

run("Erode");

run("Create Selection");

selectWindow("C:/Users/Data source/b3-2.czi - C=1");

run("Subtract Background...", "rolling=10 sliding");

run("Restore Selection");

run("Measure");

selectWindow("C:/Users/Data source/b3-2.czi - C=1");

close();

selectWindow("C:/Users/Data source/b3-2.czi - C=0");

close();

selectWindow("C:/Users/Data source/b3-2.czi - C=1-1");

close();

open("C:/Users/Data source/g1-1.czi");

run("Duplicate...", " ");

run("Subtract Background...", "rolling=10 sliding");

run("Auto Threshold", "method=Default white");

run("Convert to Mask");

run("Erode");

run("Create Selection");

selectWindow("C:/Users/Data source/g1-1.czi - C=1");

run("Subtract Background...", "rolling=10 sliding");

run("Restore Selection");

run("Measure");

selectWindow("C:/Users/Data source/g1-1.czi - C=1");

close();

selectWindow("C:/Users/Data source/g1-1.czi - C=0");

close();

selectWindow("C:/Users/Data source/g1-1.czi - C=1-1");

close();

open("C:/Users/Data source/g1-2.czi");

run("Duplicate...", " ");

run("Subtract Background...", "rolling=10 sliding");

run("Auto Threshold", "method=Default white");

run("Convert to Mask");

run("Erode");

run("Create Selection");

selectWindow("C:/Users/Data source/g1-2.czi - C=1");

run("Subtract Background...", "rolling=10 sliding");

run("Restore Selection");

run("Measure");

selectWindow("C:/Users/Data source/g1-2.czi - C=1");

close();

selectWindow("C:/Users/Data source/g1-2.czi - C=0");

close();

selectWindow("C:/Users/Data source/g1-2.czi - C=1-1");

close();

open("C:/Users/Data source/g1-3.czi");

run("Duplicate...", " ");

run("Subtract Background...", "rolling=10 sliding");

run("Auto Threshold", "method=Default white");

run("Convert to Mask");

run("Erode");

run("Create Selection");

selectWindow("C:/Users/Data source/g1-3.czi - C=1");

run("Subtract Background...", "rolling=10 sliding");

run("Restore Selection");

run("Measure");

selectWindow("C:/Users/Data source/g1-3.czi - C=1");

close();

selectWindow("C:/Users/Data source/g1-3.czi - C=0");

close();

selectWindow("C:/Users/Data source/g1-3.czi - C=1-1");

close();

open("C:/Users/Data source/HI RH1-1.czi");

run("Duplicate...", " ");

run("Subtract Background...", "rolling=10 sliding");

run("Auto Threshold", "method=Default white");

run("Convert to Mask");

run("Erode");

run("Create Selection");

selectWindow("C:/Users/Data source/HI RH1-1.czi - C=1");

run("Subtract Background...", "rolling=10 sliding");

run("Restore Selection");

run("Measure");

selectWindow("C:/Users/Data source/HI RH1-1.czi - C=1");

close();

selectWindow("C:/Users/Data source/HI RH1-1.czi - C=0");

close();

selectWindow("C:/Users/Data source/HI RH1-1.czi - C=1-1");

close();

open("C:/Users/Data source/HI RH1-2.czi");

run("Duplicate...", " ");

run("Subtract Background...", "rolling=10 sliding");

run("Auto Threshold", "method=Default white");

run("Convert to Mask");

run("Erode");

run("Create Selection");

selectWindow("C:/Users/Data source/HI RH1-2.czi - C=1");

run("Subtract Background...", "rolling=10 sliding");

run("Restore Selection");

run("Measure");

selectWindow("C:/Users/Data source/HI RH1-2.czi - C=1");

close();

selectWindow("C:/Users/Data source/HI RH1-2.czi - C=0");

close();

selectWindow("C:/Users/Data source/HI RH1-2.czi - C=1-1");

close();

open("C:/Users/Data source/HI RH1-3.czi");

run("Duplicate...", " ");

run("Subtract Background...", "rolling=10 sliding");

run("Auto Threshold", "method=Default white");

run("Convert to Mask");

run("Erode");

run("Create Selection");

selectWindow("C:/Users/Data source/HI RH1-3.czi - C=1");

run("Subtract Background...", "rolling=10 sliding");

run("Restore Selection");

run("Measure");

selectWindow("C:/Users/Data source/HI RH1-3.czi - C=1");

close();

selectWindow("C:/Users/Data source/HI RH1-3.czi - C=0");

close();

selectWindow("C:/Users/Data source/HI RH1-3.czi - C=1-1");

close();

open("C:/Users/Data source/RH1-1.czi");

run("Duplicate...", " ");

run("Subtract Background...", "rolling=10 sliding");

run("Auto Threshold", "method=Default white");

run("Convert to Mask");

run("Erode");

run("Create Selection");

selectWindow("C:/Users/Data source/RH1-1.czi - C=1");

run("Subtract Background...", "rolling=10 sliding");

run("Restore Selection");

run("Measure");

selectWindow("C:/Users/Data source/RH1-1.czi - C=1");

close();

selectWindow("C:/Users/Data source/RH1-1.czi - C=0");

close();

selectWindow("C:/Users/Data source/RH1-1.czi - C=1-1");

close();

open("C:/Users/Data source/RH1-2.czi");

run("Duplicate...", " ");

run("Subtract Background...", "rolling=10 sliding");

run("Auto Threshold", "method=Default white");

run("Convert to Mask");

run("Erode");

run("Create Selection");

selectWindow("C:/Users/Data source/RH1-2.czi - C=1");

run("Subtract Background...", "rolling=10 sliding");

run("Restore Selection");

run("Measure");

selectWindow("C:/Users/Data source/RH1-2.czi - C=1");

close();

selectWindow("C:/Users/Data source/RH1-2.czi - C=0");

close();

selectWindow("C:/Users/Data source/RH1-2.czi - C=1-1");

close();

open("C:/Users/Data source/RH1-3.czi");

run("Duplicate...", " ");

run("Subtract Background...", "rolling=10 sliding");

run("Auto Threshold", "method=Default white");

run("Convert to Mask");

run("Erode");

run("Create Selection");

selectWindow("C:/Users/Data source/RH1-3.czi - C=1");

run("Subtract Background...", "rolling=10 sliding");

run("Restore Selection");

run("Measure");

selectWindow("C:/Users/Data source/RH1-3.czi - C=1");

close();

selectWindow("C:/Users/Data source/RH1-3.czi - C=0");

close();

selectWindow("C:/Users/Data source/RH1-3.czi - C=1-1");

close();

open("C:/Users/Data source/RH2-1.czi");

run("Duplicate...", " ");

run("Subtract Background...", "rolling=10 sliding");

run("Auto Threshold", "method=Default white");

run("Convert to Mask");

run("Erode");

run("Create Selection");

selectWindow("C:/Users/Data source/RH2-1.czi - C=1");

run("Subtract Background...", "rolling=10 sliding");

run("Restore Selection");

run("Measure");

selectWindow("C:/Users/Data source/RH2-1.czi - C=1");

close();

selectWindow("C:/Users/Data source/RH2-1.czi - C=0");

close();

selectWindow("C:/Users/Data source/RH2-1.czi - C=1-1");

close();

open("C:/Users/Data source/RH2-2.czi");

run("Duplicate...", " ");

run("Subtract Background...", "rolling=10 sliding");

run("Auto Threshold", "method=Default white");

run("Convert to Mask");

run("Erode");

run("Create Selection");

selectWindow("C:/Users/Data source/RH2-2.czi - C=1");

run("Subtract Background...", "rolling=10 sliding");

run("Restore Selection");

run("Measure");

selectWindow("C:/Users/Data source/RH2-2.czi - C=1");

close();

selectWindow("C:/Users/Data source/RH2-2.czi - C=0");

close();

selectWindow("C:/Users/Data source/RH2-2.czi - C=1-1");

close();

open("C:/Users/Data source/RH2-3.czi");

run("Duplicate...", " ");

run("Subtract Background...", "rolling=10 sliding");

run("Auto Threshold", "method=Default white");

run("Convert to Mask");

run("Erode");

run("Create Selection");

selectWindow("C:/Users/Data source/RH2-3.czi - C=1");

run("Subtract Background...", "rolling=10 sliding");

run("Restore Selection");

run("Measure");

selectWindow("C:/Users/Data source/RH2-3.czi - C=1");

close();

selectWindow("C:/Users/Data source/RH2-3.czi - C=0");

close();

selectWindow("C:/Users/Data source/RH2-3.czi - C=1-1");

close();

open("C:/Users/Data source/RH3-1.czi");

run("Duplicate...", " ");

run("Subtract Background...", "rolling=10 sliding");

run("Auto Threshold", "method=Default white");

run("Convert to Mask");

run("Erode");

run("Create Selection");

selectWindow("C:/Users/Data source/RH3-1.czi - C=1");

run("Subtract Background...", "rolling=10 sliding");

run("Restore Selection");

run("Measure");

selectWindow("C:/Users/Data source/RH3-1.czi - C=1");

close();

selectWindow("C:/Users/Data source/RH3-1.czi - C=0");

close();

selectWindow("C:/Users/Data source/RH3-1.czi - C=1-1");

close();

open("C:/Users/Data source/RH3-2.czi");

run("Duplicate...", " ");

run("Subtract Background...", "rolling=10 sliding");

run("Auto Threshold", "method=Default white");

run("Convert to Mask");

run("Erode");

run("Create Selection");

selectWindow("C:/Users/Data source/RH3-2.czi - C=1");

run("Subtract Background...", "rolling=10 sliding");

run("Restore Selection");

run("Measure");

selectWindow("C:/Users/Data source/RH3-2.czi - C=1");

close();

selectWindow("C:/Users/Data source/RH3-2.czi - C=0");

close();

selectWindow("C:/Users/Data source/RH3-2.czi - C=1-1");

close();

open("C:/Users/Data source/RH3-3.czi");

run("Duplicate...", " ");

run("Subtract Background...", "rolling=10 sliding");

run("Auto Threshold", "method=Default white");

run("Convert to Mask");

run("Erode");

run("Create Selection");

selectWindow("C:/Users/Data source/RH3-3.czi - C=1");

run("Subtract Background...", "rolling=10 sliding");

run("Restore Selection");

run("Measure");

selectWindow("C:/Users/Data source/RH3-3.czi - C=1");

close();

selectWindow("C:/Users/Data source/RH3-3.czi - C=0");

close();

selectWindow("C:/Users/Data source/RH3-3.czi - C=1-1");

close();

open("C:/Users/Data source/RH-lambda1-1.czi");

run("Duplicate...", " ");

run("Subtract Background...", "rolling=10 sliding");

run("Auto Threshold", "method=Default white");

run("Convert to Mask");

run("Erode");

run("Create Selection");

selectWindow("C:/Users/Data source/RH-lambda1-1.czi - C=1");

run("Subtract Background...", "rolling=10 sliding");

run("Restore Selection");

run("Measure");

selectWindow("C:/Users/Data source/RH-lambda1-1.czi - C=1");

close();

selectWindow("C:/Users/Data source/RH-lambda1-1.czi - C=0");

close();

selectWindow("C:/Users/Data source/RH-lambda1-1.czi - C=1-1");

close();

open("C:/Users/Data source/RH-lambda1-2.czi");

run("Duplicate...", " ");

run("Subtract Background...", "rolling=10 sliding");

run("Auto Threshold", "method=Default white");

run("Convert to Mask");

run("Erode");

run("Create Selection");

selectWindow("C:/Users/Data source/RH-lambda1-2.czi - C=1");

run("Subtract Background...", "rolling=10 sliding");

run("Restore Selection");

run("Measure");

selectWindow("C:/Users/Data source/RH-lambda1-2.czi - C=1");

close();

selectWindow("C:/Users/Data source/RH-lambda1-2.czi - C=0");

close();

selectWindow("C:/Users/Data source/RH-lambda1-2.czi - C=1-1");

close();

open("C:/Users/Data source/RH-lambda1-3.czi");

run("Duplicate...", " ");

run("Subtract Background...", "rolling=10 sliding");

run("Auto Threshold", "method=Default white");

run("Convert to Mask");

run("Erode");

run("Create Selection");

selectWindow("C:/Users/Data source/RH-lambda1-3.czi - C=1");

run("Subtract Background...", "rolling=10 sliding");

run("Restore Selection");

run("Measure");

selectWindow("C:/Users/Data source/RH-lambda1-3.czi - C=1");

close();

selectWindow("C:/Users/Data source/RH-lambda1-3.czi - C=0");

close();

selectWindow("C:/Users/Data source/RH-lambda1-3.czi - C=1-1");

close();

open("C:/Users/Data source/RH-lambda2-1.czi");

run("Duplicate...", " ");

run("Subtract Background...", "rolling=10 sliding");

run("Auto Threshold", "method=Default white");

run("Convert to Mask");

run("Erode");

run("Create Selection");

selectWindow("C:/Users/Data source/RH-lambda2-1.czi - C=1");

run("Subtract Background...", "rolling=10 sliding");

run("Restore Selection");

run("Measure");

selectWindow("C:/Users/Data source/RH-lambda2-1.czi - C=1");

close();

selectWindow("C:/Users/Data source/RH-lambda2-1.czi - C=0");

close();

selectWindow("C:/Users/Data source/RH-lambda2-1.czi - C=1-1");

close();

open("C:/Users/Data source/RH-lambda2-2.czi");

run("Duplicate...", " ");

run("Subtract Background...", "rolling=10 sliding");

run("Auto Threshold", "method=Default white");

run("Convert to Mask");

run("Erode");

run("Create Selection");

selectWindow("C:/Users/Data source/RH-lambda2-2.czi - C=1");

run("Subtract Background...", "rolling=10 sliding");

run("Restore Selection");

run("Measure");

selectWindow("C:/Users/Data source/RH-lambda2-2.czi - C=1");

close();

selectWindow("C:/Users/Data source/RH-lambda2-2.czi - C=0");

close();

selectWindow("C:/Users/Data source/RH-lambda2-2.czi - C=1-1");

close();

open("C:/Users/Data source/RH-lambda2-3.czi");

run("Duplicate...", " ");

run("Subtract Background...", "rolling=10 sliding");

run("Auto Threshold", "method=Default white");

run("Convert to Mask");

run("Erode");

run("Create Selection");

selectWindow("C:/Users/Data source/RH-lambda2-3.czi - C=1");

run("Subtract Background...", "rolling=10 sliding");

run("Restore Selection");

run("Measure");

selectWindow("C:/Users/Data source/RH-lambda2-3.czi - C=1");

close();

selectWindow("C:/Users/Data source/RH-lambda2-3.czi - C=0");

close();

selectWindow("C:/Users/Data source/RH-lambda2-3.czi - C=1-1");

close();
